# Hypoxia‐induced PGK1 expression promotes esophageal squamous cell carcinoma progression via stimulating MYH9‐mediated GSK3β/β‐catenin signalling

**DOI:** 10.1002/ctm2.70376

**Published:** 2025-06-18

**Authors:** Jia‐cheng Xu, Lin‐feng Wu, Tian‐yin Chen, Yan‐bo Liu, Yi‐fei Zhang, Ping‐hong Zhou, Yi‐qun Zhang

**Affiliations:** ^1^ Department of Endoscopy Center and Endoscopy Research Institute Zhongshan Hospital Fudan University Shanghai China; ^2^ Department of Endoscopy Shanghai Collaborative Innovation Center Shanghai China

**Keywords:** β‐catenin signalling pathway, esophageal squamous cell carcinoma, hypoxia, MYH9, PGK1

## Abstract

**Background:**

Phosphoglycerate kinase 1 (PGK1) serves as a critical metabolic enzyme in the process of glycolysis and has many nonmetabolic functions in tumour progression. One of the most prevalent malignant tumours is still esophageal squamous cell carcinoma (ESCC), with high recurrence rates, high probabilities of metastasis, and poor prognoses. However, the molecular mechanisms and physiological contribution of PGK1 to ESCC carcinogenesis remain largely elusive.

**Methods:**

Esophageal cancer bioinformatics analysis and tissue microarray analysis were employed to elucidate the aberrant expression of PGK1 during ESCC progression. The carcinogenic effect of PGK1 was examined using cell proliferation, migration and sphere formation assays. Mass spectrometry analysis, immunoprecipitation, ChIP and luciferase assays, hypoxia assays and in vitro and in vivo experiments were used to clarify the mechanism of the PGK1‒MYH9 interaction in the β‐catenin/c‐Myc signalling pathway.

**Results:**

We clarified that in patients with ESCC, elevated PGK1 levels were linked to poor survival, tumour size, lymph node metastatic status, and TNM stage. In vivo and in vitro experimental analyses revealed that PGK1 promoted ESCC cell tumour stemness and EMT both in vivo and in vitro. Mechanistically, we discovered that PGK1 interacts with myosin‐9 (MYH9), leading to MYH9‐mediated ubiquitination‐mediated degradation of GSK‐3β, thereby triggering the β‐catenin signalling pathway and transcriptionally increasing c‐Myc expression. In addition, we found that hypoxic conditions upregulated PGK1, with HIF‐1α transactivating PGK1 expression, further promoting the PGK1‐MYH9 interaction and PGK1/MYH9/β‐catenin/c‐Myc axis activation.

**Conclusions:**

PGK1 promotes ESCC tumourigenicity and migratory capacity by facilitating β‐catenin‐dependent c‐Myc transcription. Under hypoxic conditions, the PGK1‒MYH9 interaction is strengthened, and HIF‐1α‐mediated transcription increases PGK1 expression, thereby activating the β‐catenin/c‐Myc signalling pathway. Taken together, PGK1 holds promise as a potential biomarker for predicting postoperative prognosis and recurrence in patients with ESCC.

## Graphical abstract

Under hypoxic conditions, elevated PGK1 interacts with MYH9, promoting the ubiquitination and degradation of GSK3β, which leads to β‐catenin stabilisation and nuclear translocation. Activated β‐catenin increases the transcription of c‐Myc and downstream targets genes enhancing cancer stemness, epithelial‐to‐mesenchymal transition (EMT), metastasis, and ESCC recurrence.

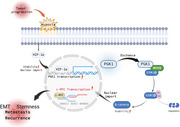

## 1 INTRODUCTION

Esophageal cancer is a prevalent malignancy of the digestive tract. Globally, it ranks 11th in incidence and 7th in mortality. In China, it holds the 6th position in incidence and the 5th in mortality, highlighting the critical significance of its highly aggressive nature.^1^, ^2^, ^3^ Esophageal squamous cell carcinoma (ESCC) represents the predominant histological subtype in China, accounting for approximately 90% of all cases.^4^ At present, early‐stage esophageal cancer can be effectively treated with minimally invasive operation like endoscopic submucosal dissection (ESD), which enables complete tumour removal while maintaining the digestive tract's structural integrity. These approaches confer superior prognostic outcomes and enhanced quality of patients’ life.^5^ However, the diagnosis of esophageal cancer is often delayed due to the insidious onset and propensity for early lymphatic dissemination. As a result, most ESCC patients receive their diagnosis at an advanced stage, necessitating esophageal and digestive tract reconstruction, which significantly reduced overall survival rate, resulting in poor postoperative prognosis.^6^, ^7^ Despite recent progress in multidisciplinary treatment approaches in ESCC therapy, including neoadjuvant chemoradiotherapy, the development of effective therapies is still limited by the lack of viable molecular targets. The overall 5‐year survival rate of ESCC patients remains at less than 15%, with the majority of patients succumbing to tumour recurrence and metastasis.^8^ These challenges disclose an urgent need to find novel therapeutic targets and elucidate the molecular mechanisms driving esophageal cancer progression, to inform the development of more effective treatment strategies.

Phosphoglycerate kinase1 (PGK1) serves as a critical metabolic enzyme in the process of glycolysis. As the first ATP‐generating enzyme in the glycolytic pathway, PGK1 catalyses the conversion of 1,3‐bisphosphoglycerate (1,3‐BPG) to 3‐phosphoglycerate (3‐PG), simultaneously producing ATP.^9^ Recent research has demonstrated that PGK1 is aberrantly activated in neoplastic cells, functions as a pivotal oncogenic factor in various tumour types.^10^, ^11^, ^12^ Furthermore, beyond its metabolic functions, PGK1 has been increasingly recognised for its non‐metabolic roles in various cellular processes, particularly in cancer progression.^13^, ^14^ PGK1's physiological function in carcinogenesis is more complicated, though, and appears to rely on the kind of tissue and the cellular environment. For instance, in pancreatic cancer, PGK1 can translocate into the nucleus and participate in the regulation of gene transcription under SMAD4 mutation, thereby inducing the invasion capabilities of pancreatic cancer cells.^15^ But in glioma, when cells are stimulated by driving factors such as K‐Ras, B‐Raf mutations, PGK1 can activate pyruvate dehydrogenase kinase 1 (PDHK1) phosphorylation, suppressing the tricarboxylic acid (TCA) cycle ultimately promoting tumour growth. Even same in gliomas, PGK1, when activated by phosphorylation of CK2α, translocated in the nucleus but interacting with CDC7, participated in DNA replication.^16^ Up to now, there have been no studies on the relationship between PGK1 and esophageal cancer, the precise roles and molecular mechanisms underlying its function in ESCC remain insufficiently elucidated.

Hypoxia constitutes an important characteristic of the tumour microenvironment (TME) in numerous solid malignancies.^17^ As tumours expand beyond the capacity of their pre‐existing vasculature, the imbalance between oxygen supply and oxygen demand culminates in the establishment of a hypoxic TME.^18^ Tumour cells, however, are not passive victims of this adverse milieu; rather, they undergo an evolutionary process to develop compensatory mechanisms that enable their survival and proliferation under hypoxic TME.^19^ Hypoxia‐inducible factor‐1 (HIF‐1), a master transcription factor in the cellular response to hypoxia, is essential to this adaptive process. HIF‐1α is the key transcriptional subunit of HIF‐1, regulating a broad spectrum of cellular processes by enhancing the expression of downstream target genes, glycolytic enzymes, transcription factors and other factors associated with epithelial‐to‐mesenchymal transition (EMT) especially. These regulatory activities collectively facilitate angiogenesis, metabolic reprogramming, and cellular adaptation to oxygen deprivation.^20^

According to this study, PGK1 is abnormally overexpressed in esophageal squamous cell carcinoma, and in patients with esophageal cancer, its expression is highly associated with a poor prognosis. Through experiments involving both the overexpression and silencing of PGK1, we elucidated its pivotal role in facilitating esophageal cancer progression, including tumour growth, metastasis, and maintenance of cancer stemness. Moreover, via mass spectrometry, we uncovered MYH9 as a critical downstream effector of PGK1. PGK1 enhances MYH9‐mediated ubiquitination of GSK3β through directly interaction with MYH9, thereby activating the β‐catenin signalling pathway, transcriptionally increased c‐Myc expression. Notably, we also observed that hypoxia induces a concomitant upregulation of PGK1 transcription and strengthens its interaction with MYH9. These strong results imply that PGK1 may be a feasible therapeutic target in esophageal cancer and offer important insights into the molecular mechanisms driving tumour hypoxia.

## 2 MATERIALS AND METHODS

### 2.1 Clinical samples

All patients provided written informed consent for the use of their information and samples in research. Tissue sample were collected from 108 esophageal cancer patients who underwent surgical resection with complete follow‐up information at Zhongshan Hospital, Fudan University (Shanghai, China).

### 2.2 Cell lines and culture

The human ESCC cell lines ECA109, KYSE140, KYSE150, KYSE510, TE‐1 and the normal esophageal epithelial cell line HET‐1A were obtained from the Endoscopy Research Institute at Fudan University. HEK293T were obtained from the Cell Bank of the Chinese Academy of Sciences. Cells were cultured in DMEM or RPMI‐1640 with 10% FBS and 1% penicillin‐streptomycin solution in a humidified atmosphere containing 5% CO_2_ at 37°C. All cell lines were routinely tested to be negative for mycoplasma contamination. For hypoxia assays, ESCC cells were cultured under hypoxic conditions using a controlled hypoxia chamber set at 1% O₂, 5% CO₂, and 94% N₂. The cells were exposed to hypoxia for 12 h before being subjected to the stemness (sphere formation) and migration assays.

### 2.3 RNA extraction, reverse transcription and qRT‐PCR

Total RNA was isolated by Trizol reagent (Invitrogen, USA) and reverse transcribed by cDNA Synthesis SuperMix as the manufacturer's instructions. The Real‐time polymerase chain reactions (qRT‐PCR) assay was performed by qPCR SYBR Green Master Mix in StepOne Plus. Primer sequences were shown in Table S4. Relative gene expression analysis was performed using the equation 2^−△△^CT was used to perform expression analysis. As an internal control, ACTB was employed.

### 2.4 Western blotting

SDS lysis buffer containing a protease and phosphatase inhibitor cocktail was used to extract the total protein. Protein concentration was measured by a BCA assay kit. After being separated on SDS‐PAGE gels, protein lysates were transferred onto PVDF membranes. The membranes were blocked with 5% non‐fat milk in TBST for 1 h. After an overnight incubation at 4°C with the primary antibody, the secondary antibody was incubated for an hour at room temperature. Antibody information was shown in Table S3. High‐sig ECL substrate was used to visualise the proteins (Tanon, China).

### 2.5 Immunohistochemical (IHC) staining

Immunohistochemical staining assay was carried out using IHC Assay Kit according to the manufacturer's protocol. Primary antibody was used to incubate the sections. Antibody information was shown in Table S3. The composite expression score (CES) was calculated from staining intensity (0, no staining; 1, weak; 2, moderate; 3, strong) and positive tumour cells area (1, 1%–25%; 2, 26%–50%; 3, 51%–75%; 4, 76%–100%). CES = intensity score × frequency score.

### 2.6 Cell proliferation assay

The Cell Counting Kit‐8 was used to measure cell proliferation, and it was carried out according to the manufacturer's instructions. 1000 cells per well were seeded into 96‐well plates. Following the first, second, third, and fourth days, 10 µL of CCK‐8 reagent was applied to each well. After 2 h of incubation, the absorbance at 450 nm was measured on the plates.

### 2.7 Migration assay

For the cell migration assay, FBS‐free media was used to seed 5 × 10^4^ of ECA109, 2 × 10^4^ KYSE150 cells in the upper chambers of Transwell Clear Polyester Membrane Inserts. The bottom chambers were filled with culture media that contained 20% FBS. Cells were stained with 0.1% crystal violet and treated with 4% paraformaldehyde after 48 h. On each filter, the number of cells in three fields was tallied.

### 2.8 Sphere formation assay

For sphere formation assay, 500 of ECA109/KYSE150 cells were seeded into a 96‐well plate with low attachment surface. The cells were cultured in 100 µL DMEM/F‐12 supplemented with 1 × B27, 20 ng/mL bFGF and 20 ng/mL EGF. The culture medium was replenished with 50 µL medium every 3 days. Number of spheres and sphere diameter were counted after 2 weeks incubation.

### 2.9 Protein half‐life detection

Six‐well plates were planted with cells at a confluence of about 80%. Cycloheximide (CHX; Sigma) was added to the culture medium at a final concentration of 20 µg/mL during a 24‐h incubation period. To prevent protein degradation, cells were collected and lysed using EBC buffer containing protease and phosphatase inhibitors (Sigma) at predetermined intervals after CHX treatment.

### 2.10 Animal studies

The animal experiments were conducted in accordance with the guidelines set forth by the Institutional Animal Care and Utilization Committee of Zhongshan hospital, Fudan University. We bought male BALB/c nude mice that were 5 weeks old from Shanghai SLAC Laboratory Animal Company. Every mouse was randomly assigned to a different group and kept in a pathogen‐free environment.

For the subcutaneous tumour model, 2 × 10⁶ indicated cells in 100 µL PBS were injected subcutaneously. Tumour volume was calculated as π/6 × length × width^2^ and measured as mentioned days in the figure. After 4 weeks, the mice were sacrificed, and the tumours were preserved in 4% paraformaldehyde for histological examination.

The tail vein was used to inject 3 × 10⁶ cells (ECA109) or 1 × 10⁶ cells (KYSE150) in 100 µL PBS for the lung metastasis model. After two months, lung tumour nodules were counted, and the metastatic area percentage was quantified using Photoshop.

For limiting‐dilution transplantation, 1 × 10⁶, 1 × 10⁵, or 5 × 10⁴ cells were injected into mice. The stem cell frequency were analysis use extreme limiting dilution assays (ELDA) assays.^21^

### 2.11 In vitro ubiquitination assay

His‐tagged ubiquitin (His‐Ub) and the indicated constructs were transfected into cells. To prevent degradation, cells were treated with 10 mM MG132 for 10 h after 24 h transfection. Following treatment, cells were lysed in buffer A (6 M guanidine‐HCl, 0.1 M Na_2_HPO_4_/NaH_2_PO_4_, and 10 mM imidazole, pH 8.0) and subjected to sonication to ensure complete lysis. The resulting lysates were incubated with nickel‐nitrilotriacetic acid (Ni‐NTA) agarose beads for 3 h at room temperature to capture His‐tagged proteins. To reduce non‐specific binding, the beads were sequentially washed twice with buffer A, twice with buffer A/TI (a solution of 1 volume buffer A and 3 volumes buffer TI), and once with buffer TI (25 mM Tris‐HCl and 20 mM imidazole, pH 6.8). The bound proteins were subsequently eluted and analysed by SDS‐PAGE, followed by immunoblotting to detect ubiquitinated proteins.

### 2.12 Data collection and bioinformatic analysis

TCGA‐ESCA and TCGA‐ESCC datasets are publicly available from TCGA data portal. The GEO database provided the GSE161533, GSE44021, GSE23400, GSE46452 and GSE37203. PGK1 expression was used to stratify the transcriptome data from those datasets into high‐PGK1 and low‐PGK1 groups. The GSEA v3.0 software was then used to conduct the gene set enrichment analysis (GSEA).

### 2.13 Chromatin immunoprecipitation assay

As directed by the kit, the ChIP assay was carried out using the ChIP Assay Kit. To create DNA fragments, cells were lysed, sonicated on ice, and cross‐linked with 1% formaldehyde. The fragments were used for immunoprecipitation with antibody against IgG or HIF‐1α. Then, the DNA fragments were eluted, purified and analysed by qRT‐PCR. The primers used are listed in Table S2.

### 2.14 Luciferase reporter assay

The reporter plasmids were transfected into indicated cells that had been seeded onto 24‐well plates, including c‐Myc binding sites, TOP‐Flash, FOP‐Flash, CBF1‐responsive elements, and HOP‐Flash. The Dual Luciferase Assay System methodology was used to measure the firefly and Renilla luciferase activity 48 h after transfection. Renilla fluorescence was used to normalise the luciferase activity.

### 2.15 Immunoprecipitation assay

A protease inhibitor cocktail and phosphatase inhibitors (phosphatase inhibitor cocktail set I and II) were added to EBC buffer (50 mM Tris‐HCl, pH 8.0, 120 mM NaCl, 0.5% NP‐40) before the cells were lysed. Centrifugation was used to clarify and quantify the cell lysates.

For immunoprecipitation, 2 µg of the particular antibody was incubated with 1 mg of total protein lysate for 4 h at 4°C with gently rotated. Subsequently, Protein‐A Sepharose beads were added to the mixture and incubated for an additional hour to capture immune complexes. The beads were washed four times with NETN buffer (20 mM Tris‐HCl, pH 8.0, 100 mM NaCl, 1 mM EDTA, and 0.5% NP‐40) to remove non‐specific binding proteins. The immunoprecipitated complexes were then eluted, separated by SDS‐PAGE, and analysed by immunoblotting. The intensity of the immunoblot bands was quantified using ImageJ software to evaluate protein interactions or modifications.

### 2.16 Statistical analysis

The mean ± standard deviation of a minimum of three separate experiments is used to represent the data. Software such as GraphPad Prism 9 and SPSS Statistics 26 were used to conduct statistical analysis. A two‐tailed Student's *t*‐test was utilised to compare differences between two groups and one‐way ANOVA test was used to compare three or more groups. Patients with esophageal cancer were analysed for survival using log‐rank (Mantel–Cox) tests and Kaplan–Meier survival curves. The threshold for statistical significance was set at *p* < .05. Significance was denoted as NS: not significant, *p < .05, ** *p* < .01, and *** *p* < .001.

## 3 RESULTS

### 3.1 PGK1 is highly expressed in esophageal cancer tissues and correlates with poor prognosis

**FIGURE 1:**
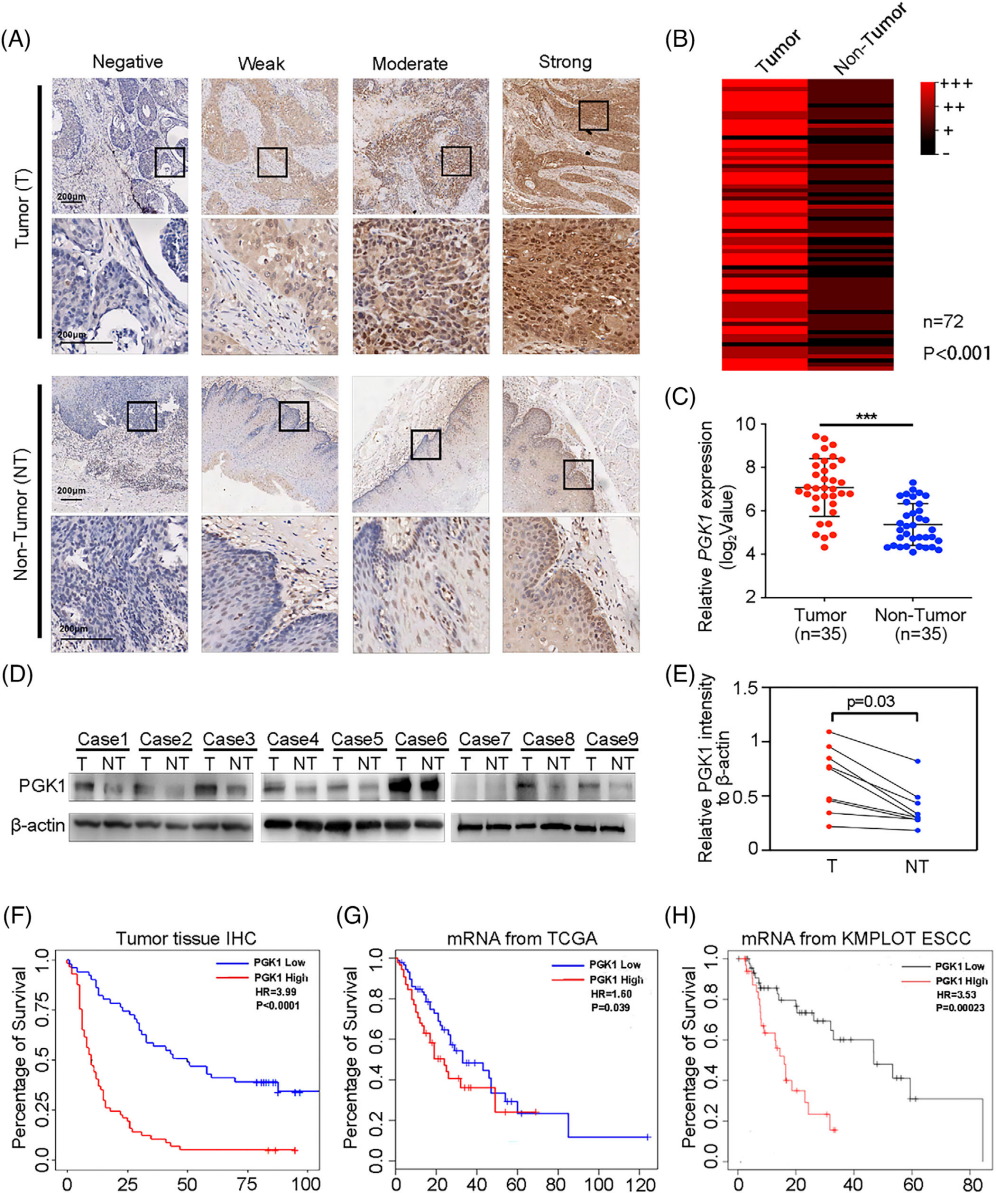
PGK1 is overexpressed in esophageal cancer and is associated with poor patient survival. (A) Immunohistochemical staining for PGK1 expression in tumour (T) and non‐tumour (NT) esophageal tissues. Representative images showing negative, weak, moderate, and strong staining intensities respectively. (B) Heatmap representing PGK1 immunohistochemistry staining intensity in 72 paired tumour and non‐tumour tissues. The intensity was scored as negative (–), weak (+), moderate (++), or strong (+++). Statistical significance was assessed using paired statistical analysis (*p* < .001). (C) Quantification of PGK1 mRNA expression (log_2_ values) in tumour and paired non‐tumour tissues (*n* = 35) by qRT‐PCR. (D) Representative immunoblotting results of PGK1 protein expression in 9 paired tumour (T) and non‐tumour (NT) tissues. β‐actin was used as the loading control. (E) Quantitative analysis of relative PGK1 protein expression normalised to β‐actin. Statistical significance was determined (*p *= .03). (F–H) Kaplan–Meier survival curves showing the overall survival of esophageal cancer patients stratified by PGK1 expression levels. (F) Survival analysis based on IHC results from tumour tissues (*n* = 108). (G) Survival analysis using data from the TCGA database (*p *= .039). (H) Survival analysis using data from the KMplot database for ESCC (*p* = .00023). Hazard ratios (HR) and *p*‐values are indicated.

To investigate the differential expression of PGK1 in esophageal cancer and non‐cancerous tissues, we first analysed the levels of PGK1 from databases. PGK1 expression in tumour tissues were higher than non‐tumour tissues from GSE161533, GSE44021, GSE23400, GSE46452, GSE37203, and TCGA database (Figure S1A–F). Immunohistochemistry was performed on 72 matched esophageal cancer tissues and peri‐tumour esophageal tissues to better illustrate the findings we obtained from the TCGA and GEO datasets. According to the research, PGK1 expression was higher in esophageal cancer than in nearby non‐tumour tissue (Figure 1A and B). Furthermore, qRT‐PCR and immunoblotting assay in esophageal cancer tissues and paired peri‐tumour esophageal tissues demonstrated that PGK1 was significantly augmented in esophageal cancer tissues (Figure 1C–E).

Next, we assessed PGK1's predictive significance in esophageal cancer. Survival analysis based on PGK1 expression data of 108 esophageal cancer patients in Zhongshan Hospital was performed. The findings showed that overall survival was noticeably poorer for those with increased PGK1 expression (Figure 1F). The data from TCGA and KMPLOT database showed consistent results (Figure 1G and H). Moreover, we found large tumour size, high T stage, high nodal stage, poor differentiation and high TNM stage were significantly correlated with high PGK1 expression in those ESCC patients (Table S1). Then, a Cox's proportional hazards model analysis of prognostic factors in those patients were performed. High PGK1 expression, male gender, poor differentiation, large tumour size, positive pathology lymph node, and high TNM stage were found associated with worse overall survival in univariate analysis. Furthermore, high PGK1 expression, and male gender were identified as the independent risk factor for worse overall survival (Table S2). These results collectively showed that PGK1 is overexpressed in tissues from esophageal cancer and is associated with a bad prognosis.

### 3.2 PGK1 promotes tumourigenicity, stemness and migration ability of ESCC cells

**FIGURE 2:**
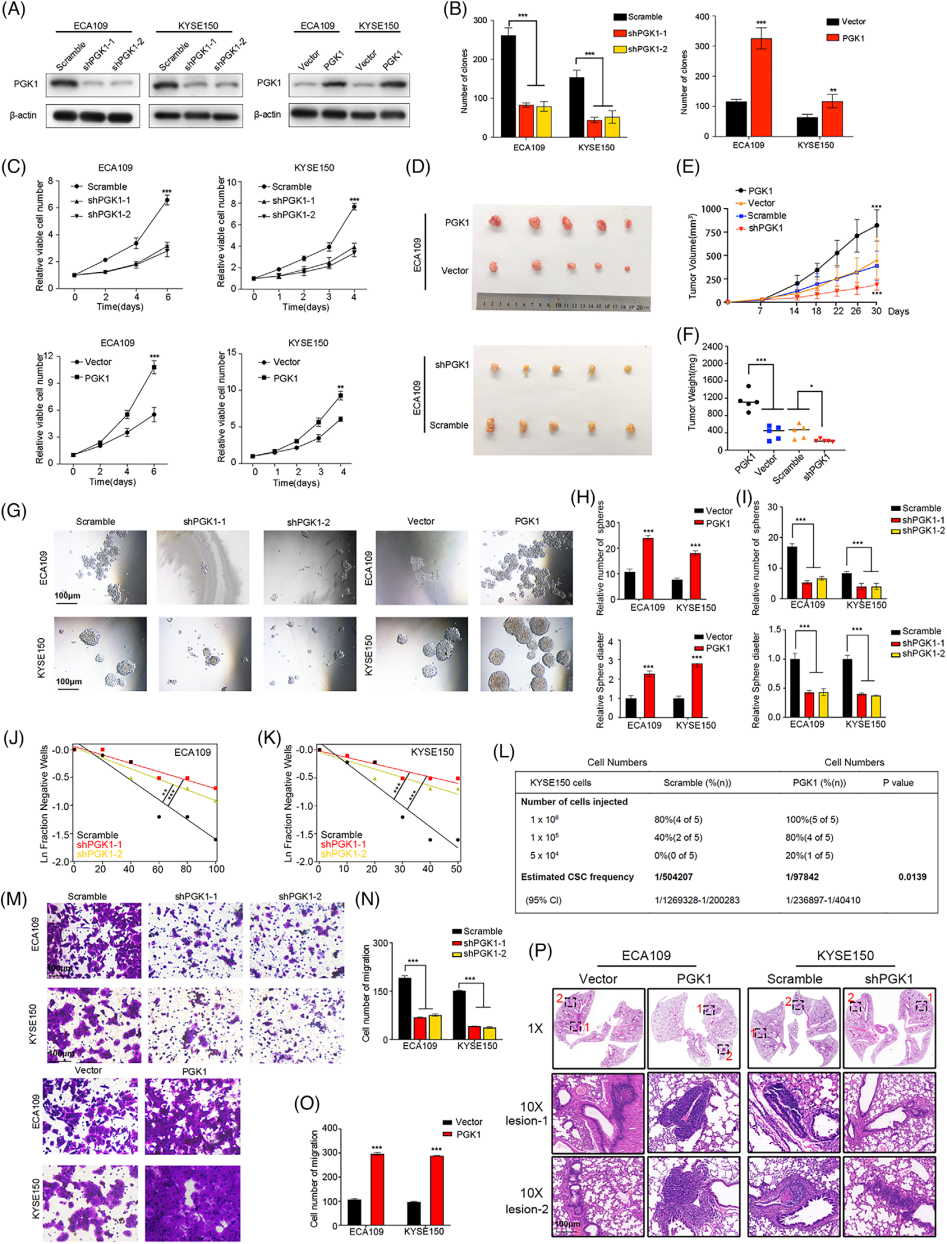
PGK1 promotes tumourigenicity, and migration ability of ESCC cells. (A) The efficiency of PGK1 knockdown and overexpression was assessed by Western Blot analysis. β‐actin served as a loading control. (B) Soft agar colony formation assay demonstrating the effect of PGK1 on colony formation in ESCC cells. (C) CCK‐8 cell viability assays showing the impact of PGK1 on ECA109 and KYSE150 cell growth over 6 days. (D–F) In vivo subcutaneous tumour xenograft models. (D) Representative images of tumours formed by ECA109 cells with PGK1 knockdown or overexpression. (E) Tumour volume measured over time. (F) Final tumour weights. (G, H) Sphere formation assays showing the effect of PGK1 knockdown (left) or overexpression (right) on the self‐renewal capacity of ECA109 and KYSE150 cells. (G) Representative images of spheres. (H, I) Quantification of the relative number of spheres. (J, K) Extreme limiting dilution assays (ELDA) estimating cancer stem cell (CSC) frequency in ECA109 (J) and KYSE150 (K) cells following PGK1 knockdown. Statistical significance is indicated. (L) Limiting dilution xenograft formation of KYSE150‐Scramble and KYSE150‐PGK1 cells in nude mice (*n* = 5 per group), including confidence intervals (CI) and *p*‐value. (M, N) Transwell migration assays demonstrating the effect of PGK1 knockdown (left) or overexpression (right) on the migration ability of ECA109 and KYSE150 cells. (M) Representative images of migrating cells. (N, O) Quantification of migration cell numbers. (P) In vivo lung metastasis models. Representative images of metastatic nodules in H&E staining of lung sections (1 × and 10 × magnifications) from mice injected with ESCC cells overexpressing or knocking down PGK1. The data from cell functional assays were presented as mean ± SD of three individual experiments, and the data from animal experiments were presented as mean ± SEM. The Student's *t*‐test was used for comparisons. *p < .05, **p < .01, ***p < .001.

We investigated PGK1 expression in a batch of esophageal cancer cell lines and esophageal normal cells to clarify the role of PGK1 in esophageal cancer. All of the esophageal cancer cells expressed more PGK1 than HET‐1A cells, according to the results of qRT‐PCR and Western blot (Figure S2A and B). We modulated the expression of PGK1 in ECA109 and KYSE150 cells via lentivirus‐mediated short‐hairpin RNAs (shPGK1‐1 and shPGK1‐2) and lentivirus‐overexpression PGK1 (Figure 2A). PGK1 overexpression resulted in significant promotion in cell proliferation, while PGK1 knockdown inhibited the cell proliferation (Figures 2B and C and S2C). Accordingly, in subcutaneous implantation nude mice models, the mice injected with ECA109‐PGK1 overexpression cells were found to have higher tumour volume and weight and Ki‐67 IHC score, whereas opposite effects were observed in mice injected with PGK1 knockdown cells, indicating that PGK1 promotes tumour tumourigenicity (Figures 2D–F and S2D and E).

Next, we verified the association between PGK1 and stemness potentials. Sphere formation assays and in vitro limited dilution assays indicated that PGK1 significantly increased the sphere formation capacity (Figure 2G–K). We created xenograft models and used extreme limiting dilution analysis (ELDA) software to measure the frequency of CSCs in order to confirm the stemness‐promoting effects of PGK1 in vivo, increased frequency of CSCs in mice after PGK1 overexpression cells injected (Figure 2L). Metastasis potential is acknowledged as one of stemness‐related features. Transwell assays indicated that PGK1 knockdown attenuated the migration of ECA109 and KYSE150 cells, and PGK1 overexpression significantly elevated the cell number of migration (Figure 2M–O). In vivo, the results of tail vein injection assay revealed that PGK1 knockdown significantly reduced the number of lung metastatic nodules, while the nodules increased when PGK1 was overexpressed (Figures 2P and S2F). Furthermore, the expression of stemness and EMT indicators was assessed using the Western blot assay and qRT‐PCR. NANOG, SOX2, SNAIL, N‐cadherin were significantly downregulated and E‐cadherin was upregulated in PGK1 knockdown cells). The converse results were found in PGK1 overexpression cells. The expression of CD44 and CD133 remained unchanged (Figure S2G–J). In summary, studies conducted both in vitro and in vivo demonstrated the critical function of PGK1 in promoting ESCC cell migration, proliferation, and stemness.

### 3.3 PGK1 upregulated the transcription of c‐Myc by activating β‐catenin signalling enhances tumour stemness an metastasis ability of ESCC cells

**FIGURE 3:**
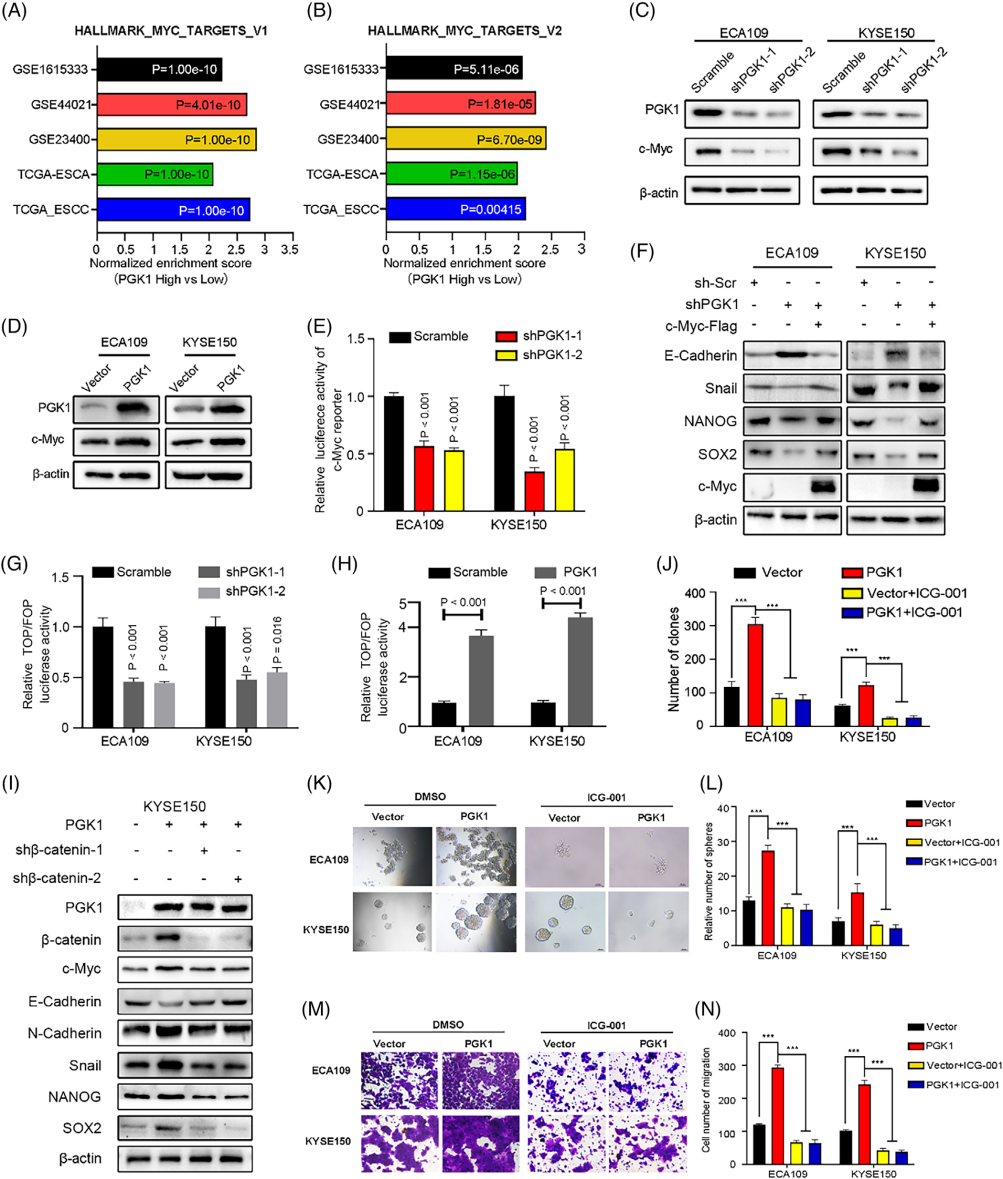
PGK1 promotes ESCC cells stemness and EMT through enhancing the transcription of c‐Myc via β‐catenin pathway. (A, B) Gene Set Enrichment Analysis (GSEA) results showing enrichment of Hallmark_MYC_Targets_V1 (A) and Hallmark_MYC_Targets_V2 (B) in esophageal cancer patients with high PGK1 expression from GSE161533, GSE44021, GSE23400, TCGA‐ESCA, and TCGA‐ESCC datasets. Normalised enrichment scores (NES) and *p*‐values are indicated. (C, D) Western blot analysis of PGK1 and c‐Myc protein levels in ECA109 and KYSE150 cells following PGK1 knockdown (shPGK1‐1 and shPGK1‐2) (C) or overexpression (D). (E) Dual‐luciferase reporter assay of c‐Myc transcriptional activity in ECA109 and KYSE150 cells with PGK1 knockdown. (F) Rescue assay with Western blot analysis of EMT and stemness markers (E‐cadherin, Snail, NANOG, SOX2) in ECA109 and KYSE150 cells. PGK1 knockdown effects were rescued by c‐Myc overexpression. (G, H) Luciferase reporter activity of TOP/FOP‐Flash assays measuring β‐catenin transcriptional activity in ECA109 and KYSE150 cells with PGK1 knockdown (G) or overexpression (H). (I) Western blot analysis of β‐catenin pathway and EMT/stemness markers in KYSE150‐PGK1 and knockdown of β‐catenin (shβ‐catenin‐1 and shβ‐catenin‐2). (J–N) Functional assays of β‐catenin activity in ECA109 and KYSE150 cells with PGK1 overexpression and treated with the β‐catenin inhibitor ICG‐001. (J) Soft agar colony formation assay. (K, L) Sphere formation assay showing representative images (K) and quantification (L). (M, N) Transwell migration assay showing representative images (M) and quantification (N). Data are presented as mean ± SD. The Student's *t*‐test was used for comparisons. *** *p* < .001.

We next investigate how PGK1 regulates ESCC cells. The gene expression patterns of patients with esophageal cancer from GSE161533, GSE44021, GSE23400, TCGA‐ESCA and TCGA‐ESCC were grouped in order to perform the GSEA. Results showed that high PGK1 expression was significantly enriched in Hallmark_Myc_Targets_V1 and Hallmark_Myc_Targets_V2 in all databases (Figure 3A and B). One of the Myc members, c‐Myc, is an oncogene in the majority of human malignancies and is essential for the regulation of stemness and EMT. Under normal conditions, the transcription, post‐transcriptional, and post‐translational processes of the proto‐oncogene c‐Myc are tightly regulated. Western blot analysis confirmed that c‐Myc protein levels significantly decreased in PGK1 knockdown cells, while opposite effects were observed in PGK1 overexpression cells (Figure 3C and D). However, PGK1 knockdown did not influence the stability of c‐Myc mRNA or protein (Figure S3B–E). PGK1 knockdown cells consistently showed a decrease in c‐Myc mRNA levels and transcriptional activity as assessed by qRT‐PCR and the dual‐luciferase reporter assay (Figures S3A and 3E). Rescue assay confirmed that c‐Myc overexpression restored the changes of biomarker protein levels by PGK1 knockdown in ESCC cells, indicating PGK1 promotes EMT and stemness through c‐Myc regulation (Figure 3F). Previous studies have reported Notch, β‐catenin and YAP pathways as the upstream regulators of c‐Myc during stemness regulation.^22^, ^23^ The results showed that transcriptional activity of YAP and Notch pathway were not affected in PGK1 knockdown cells, as indicated by HOP‐Flash and CBF1 luciferase reporter analysis respectively (Figure S3F and G). Alternatively, luciferase reporter containing TCF/LEF binding sites analysis confirmed that PGK1 activates the β‐catenin pathway (Figure 3G and H). Rescue assay demonstrated that silencing of β‐catenin restored the regulation of c‐Myc and downstream biomarker expression by PGK1 overexpression in ESCC cells (Figures 3I and S3H). Furthermore, PGK1 overexpression‐induced migration, sphere formation, and soft agar colony formation were all inhibited by the β‐catenin inhibitor ICG‐001 (Figures 3J–N and S3I). Besides, PGK1 metabolic enzyme inhibitors CBR‐470‐1 did not affect the expression of β‐catenin, c‐Myc and stemness‐related biomarkers, indicating the β‐catenin/c‐Myc pathway promoted by PGK1 was not in a metabolic enzyme‐dependent way (Figure S3J–L). However, PGK1 inhibitor‐NG52, which inhibits its activity, could suppress ESCC progression in vivo (Figure S3M–O). Collectively, our results suggest that PGK1 promotes transcriptional activity c‐Myc through β‐catenin activation in ESCC cells.

### 3.4 PGK1 activates GSK3β/β‐catenin/c‐Myc signalling pathway through interaction with MYH9

**FIGURE 4:**
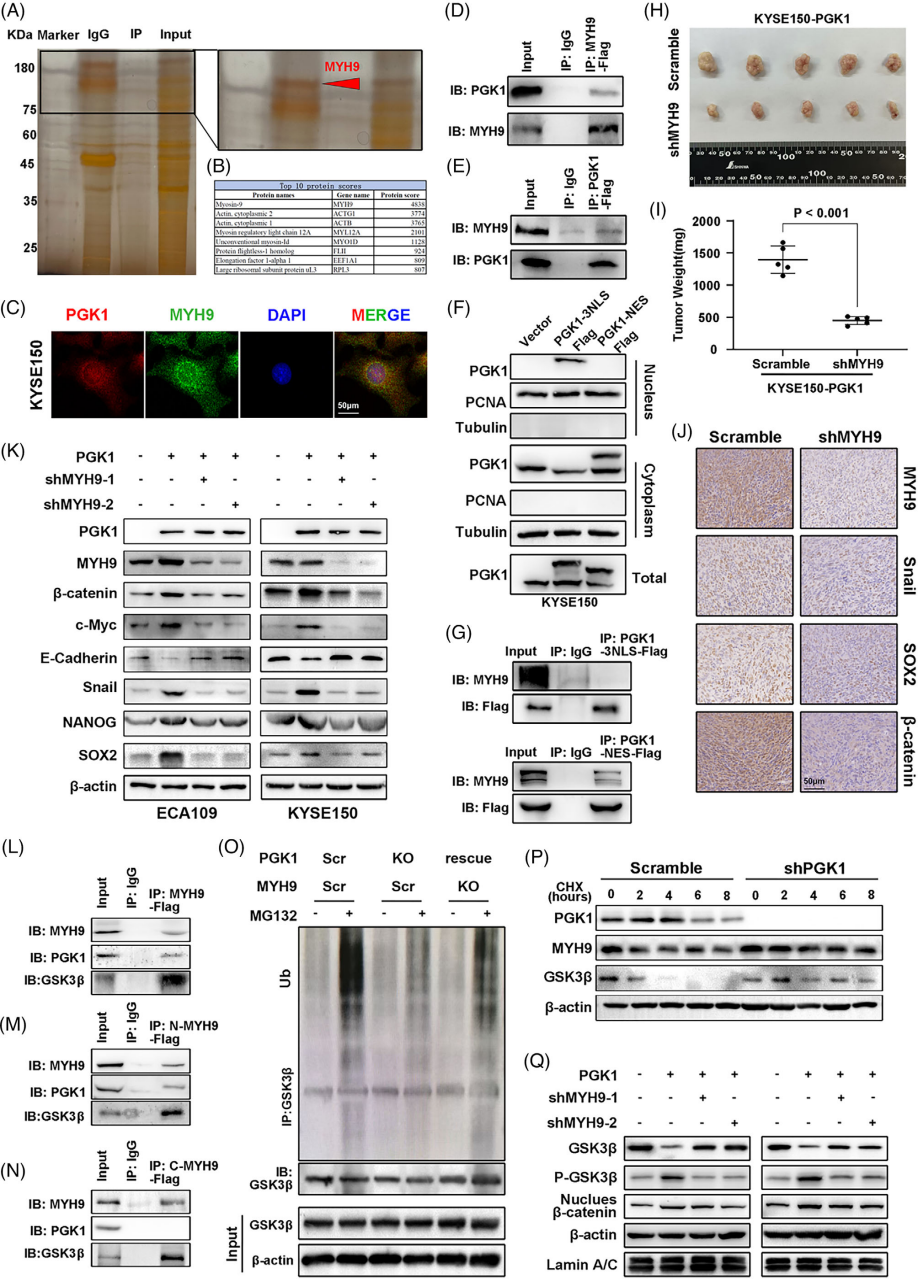
PGK1 activates the β‐catenin signalling pathway through interaction and stimulating MYH9‐mediated GSK3β ubiquitination. (A) Co‐immunoprecipitation (Co‐IP) followed by mass spectrometry identifies MYH9 as a PGK1‐interacting protein in KYSE150 ESCC cells. Red arrow indicates MYH9. (B) Table showing the top 10 proteins interacting with PGK1 based on protein scores. (C) Immunofluorescence staining shows colocalisation of PGK1 (red) and MYH9 (green) in KYSE150 cells. Nuclei were stained with DAPI (blue). (D, E) Co‐IP validation of PGK1 and MYH9 interaction in KYSE150 cells. Reciprocal IPs were performed with Flag antibodies against MYH9‐Flag or PGK1‐Flag. (F) Nuclear and cytoplasmic fractionation followed by Western blot analysis showing PGK1‐3NLS‐Flag and PGK1‐NES‐Flag localisation in KYSE150 cells. (G) Co‐IP analysis of PGK1‐3NLS‐Flag and PGK1‐NES construct confirms its interaction with MYH9. (H, I) In vivo tumourigenicity assay using KYSE150‐PGK1 cells with or without MYH9 knockdown. (H) Representative images of tumours. (I) Quantification of tumour weights (*p* < .001). Data are presented as mean ± SD. (J) Immunohistochemical analysis of MYH9, SNAIL, SOX2, and β‐catenin expression in tumours from KYSE150‐PGK1 cells with MYH9 knockdown. (K) Western blot analysis of β‐catenin, c‐Myc, EMT, and stemness markers in ECA109 and KYSE150 cells after MYH9 knockdown with or without PGK1 overexpression. (L–N) Co‐IP showing that PGK1 interacting with MYH9 and GSK3β forms a complex (L). IPs of PGK1 and N‐terminal (N‐MYH9) or C‐terminal (C‐MYH9) of MYH9‐Flag in KYSE150 cells. (O) Ubiquitination of GSK3β in KYSE150 cells with or without PGK1 and MYH9. MG132 was used to inhibit proteasomal degradation. (P) Cycloheximide (CHX) chase assay showing MYH9 and GSK3β protein stability after PGK1 knockdown in KYSE150 cells. (Q) Western blot analysis of GSK3β, phosphorylated GSK3β (P‐GSK3β), and nuclear β‐catenin in ECA109 and KYSE150 cells after MYH9 knockdown with or without PGK1 overexpression.

To elucidate the strategies by which PGK1 supports the β‐catenin/c‐Myc pathway, we employed mass spectrometry and co‐immunoprecipitation to discover proteins that interact with PGK1. Based on protein scores, we discovered that PGK1 interacted with MYH9, a GSK3β negative regulator (Figure 4A and top 1 in Figure 4B). The colocalisation of PGK1 and MYH9 in ESCC cells was confirmed by immunofluorescence assay (Figures 4C and S4A), and the interaction of two proteins was further confirmed by co‐immunoprecipitation (Figure 4D and E).

Furthermore, after exogenous transfection of nuclear‐localised PGK1 (PGK1‐3NLS) and PGK1 without nuclear localisation (PGK1‐NES), nuclear‐cytoplasmic fractionation assay indicated that PGK1‐MYH9 interaction took place in the cytoplasm (Figures 4F–G and S4B and C). To validate the effect of MYH9, we found in TCGA cohort that MYH9 had a positive correlation with GSK3B, CTNNB1, CCND1, MYC, VIM, CDH2 and SOX2 (Figure S4D). Survival analysis based on KMPLOT ESCC and ESCA cohort demonstrated patients with high MYH9 mRNA expression had better overall survival (Figure S4E and F). However, our results from IHC showed that higher MYH9 protein expression significantly correlated with worse overall survival, indicating high MYH9 protein expression, but not mRNA expression, may contributes to poor prognosis of ESCC (Figure S4G).

In vivo, xenograft model indicated that knockdown of MYH9 significantly inhibited the PGK1‐mediated tumour growth and the expression of Snail, SOX2 and β‐catenin (Figure 4H–J). In vitro, the results of western blot analysis showed that knockdown of MYH9 abrogated the alteration of the expression of β‐catenin/c‐Myc pathway biomarkers regulated by PGK1‐overexpression (Figure 4K), and there was no obvious effect of PGK1 overexpression on MYH9 expression in esophageal cancer cells (Figure S4H).

Since MYH9 activates β‐catenin pathway via promoting GSK3β‐mediated β‐catenin stability,^24^ we investigated whether PGK1 promotes the β‐catenin/c‐Myc pathway by enhancing the ubiquitination of GSK3β regulated by MYH9. Co‐immunoprecipitation assay indicated that PGK1 forms complexes with N‐terminal, instead of C‐terminal, of MYH9 with GSK3β in ESCC cells (Figure 4L–N). We next explored whether PGK1 regulated the ubiquitination and degradation of GSK3β by MYH9. The results showed that PGK1 knockdown prolonged the GSK3β protein half‐life, and reduced MYH9‐mediated GSK3β ubiquitination (Figure 4O and P) Western blot analysis indicated that silencing MYH9 abrogated the upregulated GSK3β, phosphorated‐GSK3β and nucleus β‐catenin expression by PGK1 in ESCC cells (Figure 4Q). These findings show that PGK1 interacts with MYH9 to trigger GSK3β/β‐catenin signalling, which in turn promotes tumour EMT and stemness and PGK1‐mediated cancer c‐Myc transcription.

### 3.5 Hypoxia transcriptionally regulates PGK1 via HIF‐1α and enhances PGK1‐MYH9 interaction in ESCC

**FIGURE 5:**
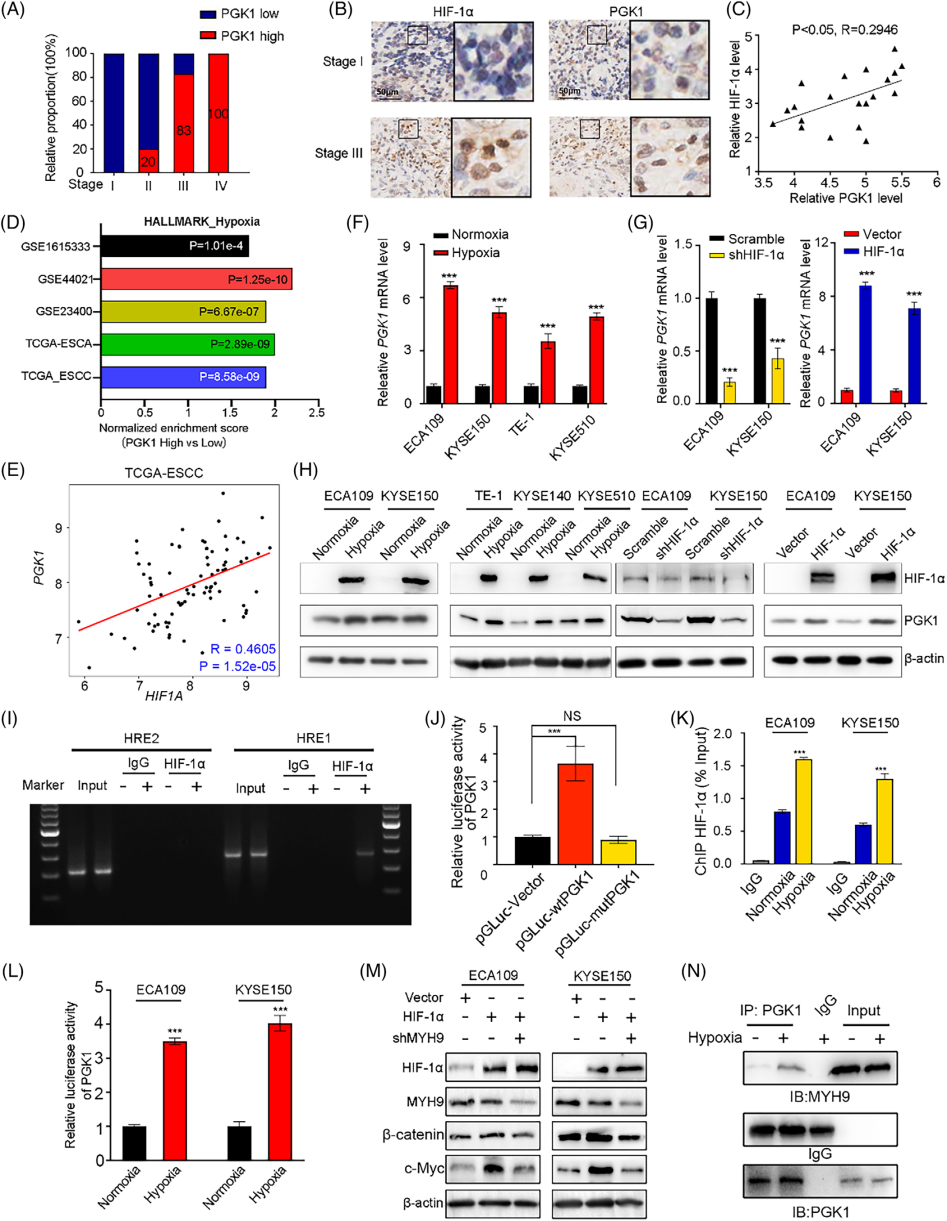
Hypoxia transcriptionally regulates the expression of PGK1 via HIF‐1α, further enhancing its interacting with MYH9. (A) Proportion of ESCC patients with high or low PGK1 expression stratified by tumour stage (I–IV). (B) Immunohistochemical staining for HIF‐1α and PGK1 in representative ESCC tissues from Stage I and Stage III patients. (C) Correlation analysis between HIF‐1α and PGK1 protein levels in esophageal cancer tissues (*p* < .05, *R* = 0.2946). (D) Gene Set Enrichment Analysis (GSEA) of Hallmark_Hypoxia signature in ESCC with high and low PGK1 expression across multiple datasets (GSE161533, GSE44021, GSE23400, TCGA‐ESCA, TCGA‐ESCC). Normalised enrichment scores (NES) and *p*‐values are shown. (E) Positive correlation between HIF‐1A and PGK1 mRNA levels in TCGA‐ESCC (*p* = 1.52e‐05, *R* = 0.4605). (F, G) qRT‐PCR analysis of PGK1 mRNA expression under normoxia or hypoxia (F) and following HIF‐1α knockdown or overexpression (G) in ESCC cell lines. Data are presented as mean ± SD; *** *p* < .001. (H) Western blot analysis of PGK1 protein expression under normoxia or hypoxia, or after HIF‐1α knockdown or overexpression. (I, J) Chromatin immunoprecipitation (ChIP) assay showing HIF‐1α binding to hypoxia‐responsive elements in the PGK1 promoter region under hypoxia. (I) Representative gel images. (J) Dual‐luciferase reporter assay showing transcriptional activity of wild‐type PGK1 promoter (pGLUC‐wtPGK1) or mutated PGK1 promoter (pGLUC‐mutPGK1) under normoxia or hypoxia. Data are presented as mean ± SD; ***p < .001, NS = not significant. (K) Quantification of ChIP enrichment (% input). Data are presented as mean ± SD; ***p < .001. (L) Dual‐luciferase reporter assay of PGK1 activity under normoxia or hypoxia in ECA109 and KYSE150 cells. Data are presented as mean ± SD; ***p < .001. (M) Western blot analysis of HIF‐1α, MYH9, β‐catenin, and c‐Myc protein with or without HIF‐1α or MYH9 knockdown. (N) Co‐immunoprecipitation assay showing interaction between PGK1 and MYH9 under hypoxia.

Performing immunohistochemistry analysis in 108 esophageal cancer tissues, we discovered that advanced esophageal cancer tissues (Stage III and IV) had noticeably greater levels of PGK1 expression than that in early phase esophageal cancer (Stage I‐II) (Figure 5A). In advanced cancer, hypoxia is a significant factor. Since it has been recognised that hypoxia‐inducible factor 1α (HIF‐1α) is a master regulator of oxygen homeostasis,^25^ we wondered if HIF‐1α could regulate PGK1 expression. Immunohistochemistry staining for PGK1 and HIF‐1α protein was performed on consecutive sections of clinical samples from Stage I and Stage III esophageal cancer patients. The observation revealed higher expression levels of both PGK1 and HIF‐1α in the tissues of Stage III patients (Figure 5B). Immunohistochemistry scoring for PGK1 and HIF‐1α in ESCC samples from microarray showed a significant correlation between HIF‐1α and PGK1 expression (Figure 5C). The consistent results were also found in the bioinformatic analysis. The results showed that high PGK1 expression group was enriched in hypoxia pathway in all databases (Figure S5A–E). PGK1 levels was significantly higher in high HIF‐1α expression group in GSE161533, GSE44021, TCGA‐ESCA and TCGA‐ESCC (Figures S5F–H and S5J), and PGK1 expression had positive correlations with HIF‐1α (Figures S5K–N and 5E). Then qRT‐PCR and Western blot assays were conducted to verify the regulatory relationship between PGK1 and HIF‐1α. We discovered that either hypoxia or HIF‐1α overexpression significantly increased PGK1 expression, and that hypoxia‐induced PGK1 upregulation was reversed when HIF‐1α was silenced (Figure 5F–H).

We used the JSAPAR platform to predict two hypoxia‐responsive elements (HRE) with the HIF‐1α‐binding consensus sequences HRE1 and HRE2 in order to verify if PGK1 is a direct transcriptional target of HIF‐1α (Figure S6). Subsequently, chromatin immunoprecipitation (ChIP) assay revealed the presence of DNA fragments containing the PGK1 promoter region HRE1 rather than HRE2 (Figure 5I), indicating HIF‐1α specifically binds to the DNA sequence HRE1 in the PGK1 promoter region. Furthermore, luciferase promoter reporter vectors were created by cloning sequences that contained either the region with mutant binding site (Mut) or the 5′‐promoter sections of PGK1(WT). The activity of promoter WT was significantly increased in hypoxic cells, while it was decrease in Mut group, suggesting the predicted HRE in the PGK1 promoter region is functional (Figure 5J). Anti‐HIF‐1α antibody‐immunoprecipitated DNA fragments from ESCC cells in hypoxia were enriched in comparison to the normoxic and IgG groups (Figure 5K). Luciferase assay confirmed hypoxia enhanced the binding of HIF‐1α and GPK1 promoter region (Figure 5J). Subsequently, the results of western blot revealed that hypoxia enhanced the interaction of PGK1 and MYH9 and promoted the MYH9/β‐catenin/c‐Myc pathway (Figure 5L–N). These results showed that in a hypoxic microenvironment, PGK1 was a direct transcriptional target of HIF‐1α.

### 3.6 . PGK1 knockdown can abrogate hypoxia‐induced ESCC tumourigenicity, tumour sphere growth and metastasis

**FIGURE 6:**
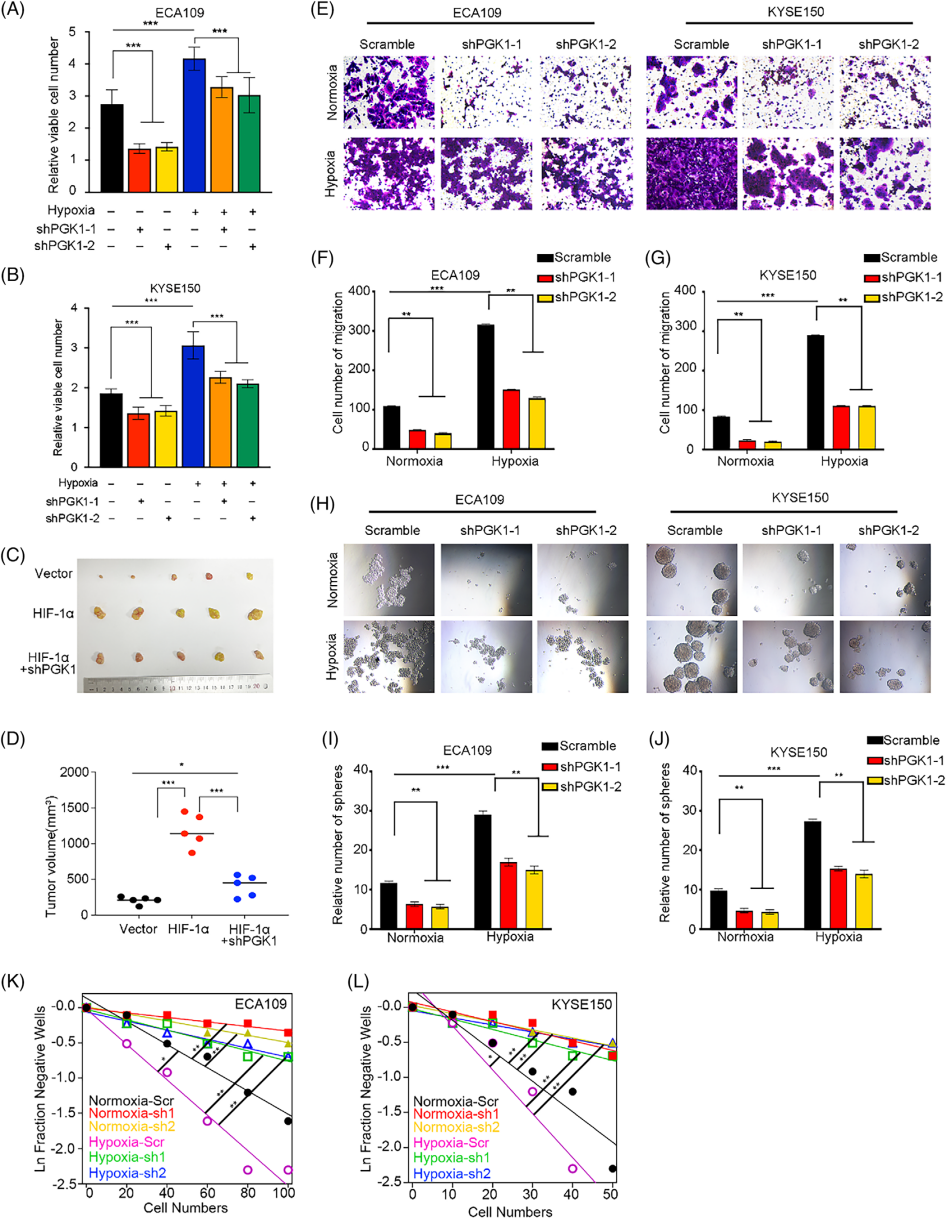
PGK1 knockout can abrogate hypoxia‐induced tumour stemness and migration in ESCC. (A, B) CCK‐8 cell viability assays showing the effects of PGK1 knockdown on hypoxia‐induced proliferation in ECA109 (A) and KYSE150 (B) cells. (C, D) In vivo tumour growth assay in nude mice injected with KYSE150 cells overexpressing HIF‐1α with or without PGK1 knockdown. (C) Representative images of tumours. (D) Quantification of tumour volumes. (E–G) Transwell migration assays demonstrating the effects of PGK1 knockdown on hypoxia‐induced migration in ECA109 and KYSE150 cells. (E) Representative images of migrating cells. (F, G) Quantification of migrating cell numbers. (H–J) Sphere formation assays showing the effects of PGK1 knockdown on hypoxia‐induced sphere‐forming ability in ECA109 and KYSE150 cells. (H) Representative images of spheres. (I, J) Quantification of relative sphere numbers. (K, L) Extreme limiting dilution assays (ELDA) estimating the frequency of cancer stem cells (CSCs) in ECA109 (K) and KYSE150 (L) cells under normoxia and hypoxia with PGK1 knockdown. The data from cell functional assays were presented as mean ± SD of three individual experiments, and the data from animal experiments were presented as mean ± SEM. The Student's *t*‐test was used for comparisons. *p < .05, **p < .01, ***p < .001.

To further explore the regulatory effect of PGK1 in ESCC cells under hypoxia condition, we then conducted CCK8, transwell, tumour sphere, and ELDA assays on ESCC cells after hypoxia treatment. It was observed that the proliferation, migration capacity of ESCC cells was increased under hypoxia compared with the normoxic treatment, thereby facilitating the formation of tumour spheres and elevating the rates of tumour formation. Subsequently, ESCC cells with PGK1 knockdown were exposed to hypoxia condition, and the above experiments were conducted again. CCK8 assays validated that knockdown of PGK1 significantly alleviated the promotion of cell proliferation (Figure 6A and B). Compared to HIF‐1α overexpression nude animals, xenograft development was much slower in mice with HIF‐1α overexpression but PGK1‐depleted cells (Figure 6C and D). Transwell assays revealed that hypoxia's promotion of tumour migration ability was abrogate by PGK1 knockdown (Figure 6E–G). Tumour sphere formation assays demonstrated that the hypoxia‐induced enhancement of ESCC cells stemness was abolished following PGK1 knockout (Figure 6H–J). Comparable outcomes were observed in ELDA assays (Figure 6K and L). Collectively, the results suggested that hypoxia tumour microenvironment promotes cell proliferation, stemness and metastasis via transcriptionally regulating PGK1 expression in esophageal cancer.

### 3.7 Correlation of PGK1 with it signalling molecules and clinical progression in ESCC

**FIGURE 7:**
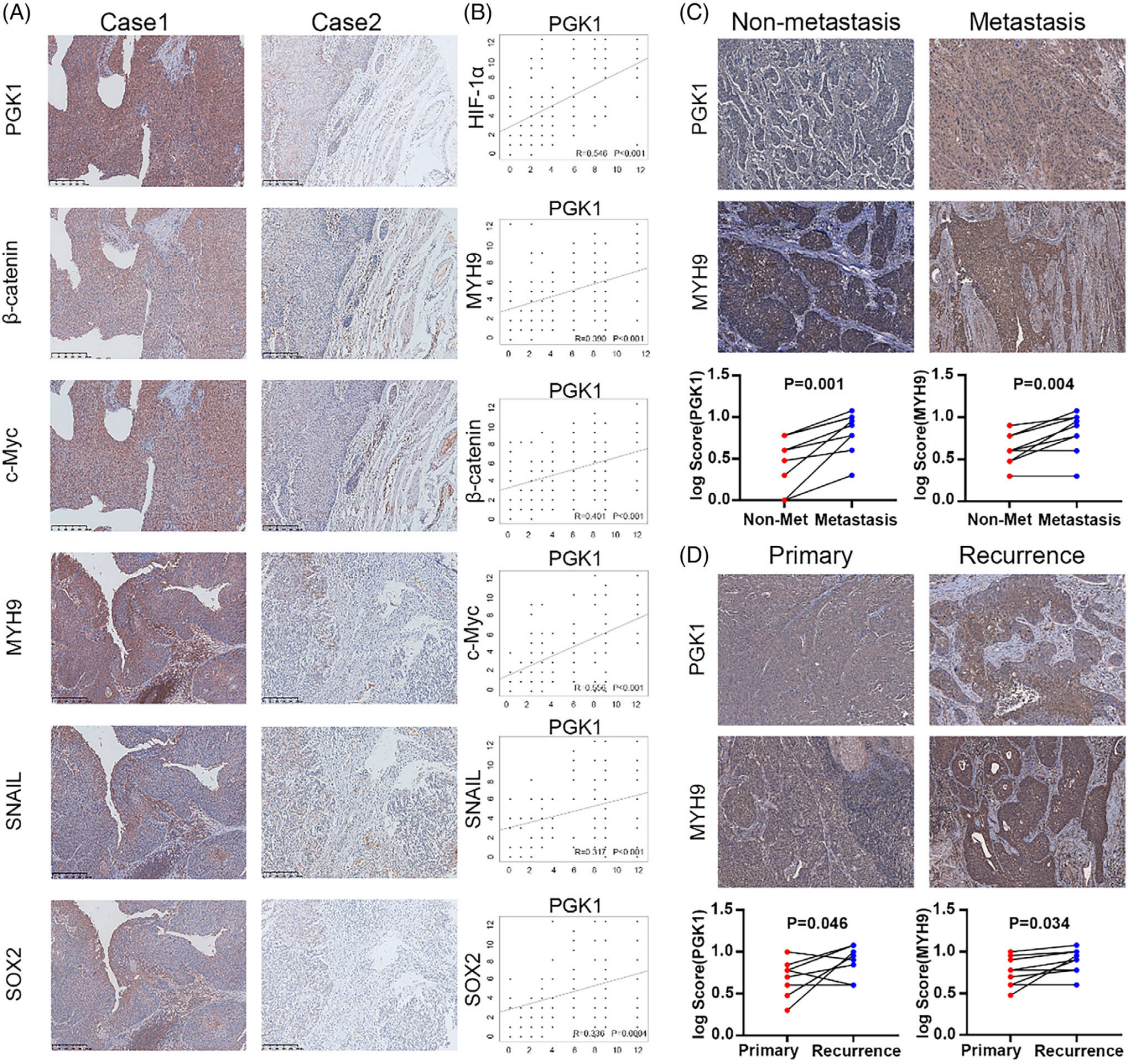
Expression correlation between PGK1 and its target genes in ESCC clinical samples. (A) Immunohistochemical analysis of PGK1, β‐catenin, c‐Myc, MYH9, SNAIL, and SOX2 expression in esophageal cancer tissues from representative cases (*n* = 88). (B) Correlation analysis between PGK1 and HIF‐1α, MYH9, β‐catenin, c‐Myc, SNAIL, and SOX2 expression levels in esophageal cancer tissues (*n* = 88). Pearson correlation coefficients (*R*) and *p*‐values are indicated. (C) Immunohistochemical staining of PGK1 and MYH9 in non‐metastatic and metastatic esophageal cancer tissues (*n* = 9). Quantification of log scores for PGK1 and MYH9 expression demonstrates significantly higher expression in metastatic tissues. Data are presented as mean ± SD; *p* = .001 (PGK1), *p* = .004 (MYH9). (D) Immunohistochemical analysis of PGK1 and MYH9 expression in primary and recurrent esophageal cancer tissues (*n* = 9). Quantification of log scores shows significantly higher expression of PGK1 and MYH9 in recurrent tissues. Data are presented as mean ± SD; *p* = .046 (PGK1), *p* = .034 (MYH9).

To validate the correlation between PGK1 and HIF‐1α, MYH9/β‐catenin/c‐Myc pathway and stemness biomarkers, immunohistochemical analysis in 88 esophageal cancer tissues from representative cases was carried out. The findings demonstrated a strong positive connection between PGK1 expression and HIF‐1α, MYH9, β‐catenin, c‐Myc, SNAIL and SOX2 (Figure 7A and B). Considering the association of cancer stemness with metastasis and recurrence, we investigated the expression of PGK1 and MYH9 in recurrent and metastatic ESCC tissues. The findings demonstrated that, in contrast to non‐metastatic tumours, both PGK1 and MYH9 were substantially expressed in metastatic tissues (Figure 7C), and in recurrent tissues compared to primary lesions (Figure 7D). The above results indicated that PGK1 expression positively correlates with HIF‐1α, MYH9/β‐catenin/c‐Myc pathway and tumour progression in ESCC clinical samples.

## 4 DISCUSSION

In this work, by analysing esophageal cancer specimen, PGK1 is highly expressed in esophageal cancer tissues across multiple cohorts and positively correlates with the size, invasion depth, lymph node metastasis, differentiation, TNM stage and poor prognosis, as a carcinogenic factor in ESCC. Furthermore, multivariate analysis revealed PGK1 as an independent factor of prognosis in ESCC. Therefore, the specific oncogenic function and physiological contribution to ESCC carcinogenesis of PGK1 in ESCC deserves to be further explored.

Here, we showed that PGK1 increases ESCC cells tumourigenicity, and promotes EMT and tumour metastasis ability. Through a biological process known as EMT, epithelial tumour cells improve their motility and invasion abilities while losing their polarity and intercellular adhesion. This transformation facilitates the detachment of tumour cells from the primary tumour mass, leading to tumour recurrence and metastasis.^26^ Additionally, EMT has been shown can augment the population of cancer stem cells (CSCs) and increase their stem‐like properties in various tumour types.^27^ Because of their capacity to self‐renew and develop, CSCs – a microscopic subgroup of cancer cells – can cause tumour recurrence, metastasis, and therapy resistance.^28^ It has been discovered that a number of chemicals and signalling pathways are essential for increasing stemness in ESCC.^29^, ^30^, ^31^ In this study, PGK1‐mediated EMT and stemness were found to be influenced by transcriptional upregulation of the oncogenic factor c‐Myc, according to GSEA and validation studies. Notch, β‐catenin and YAP pathways have been reported as the upstream regulators of c‐Myc.^22^, ^23^ Here, in ESCC cells, we identified PGK1 promotes the transcriptional expression of c‐Myc by activating the β‐catenin signalling pathway, further enhancing the transcription of EMT and stemness‐related marker, thereby driving tumour cell progression and metastasis. Interestingly, inhibition of PGK1 glycolytic activity did not markedly affect the transcriptional activity of β‐catenin/c‐Myc signalling.

By mass spectrometry analysing, we found that PGK1 interacted with MYH9 (top 1 in Figure 4B), and this interaction occurs exclusively in the cytoplasm. The heavy chain of non‐muscle myosin IIA, or MYH9, is a pivotal component of the cytoskeleton and involved in numerous cellular processes that such as cell migration division and morphology.^32^ Recent research has indicated that interaction between MYH9 and GSK‐3β promotes the ubiquitination degradation of GSK‐3β, thereby enhancing the stabilisation β‐catenin.^24^ In our work, we further demonstrated that PGK1 directly binds to the N‐domain of MYH9, promoted GSK‐3β‐mediated nuclear translocation of β‐catenin and c‐Myc transcription, indicating that these domains may play a crucial role in promoting MYH9‐mediated GSK‐3β ubiquitination.

Hypoxia is a significant feature of the tumour microenvironment in all solid tumours. However still poses significant challenges for targeted cancer therapies. In our study, we found PGK1 was upregulated in advanced tumour tissues which underwent much more hypoxia and HIF‐1α expression. Furthermore, we demonstrated that HIF‐1α transcriptionally regulated PGK1 expression, promoting PGK1‐MYH9 interaction and PGK1/MYH9/β‐catenin/c‐Myc axis activation. Hypoxia's promotion of tumourigenicity and tumour migration ability was abrogated by PGK1 knockdown, at least in the esophageal squamous cell carcinoma setting. However, the majority of tumour‐related experimental studies are conducted under normoxic conditions, which fail to replicate the profound hypoxia inherent to the in vivo TME.^33^ Even when in vitro hypoxic cultures are employed, they significantly exceed the oxygen levels encountered in the hypoxic cores of solid tumours, where oxygen deprivation is far more severe.^33^ Moreover, the degree of hypoxia varies markedly across individual tumours, underscoring the heterogeneity of hypoxic responses among malignant cells.^34^ In breast cancer, hypoxia‐induced PGK1 lysine crotonylation (Kcr) coordinate glycolysis and TCA cycle, contributing to breast cancer progression.^35^ In glioma, when cells are stimulated by hypoxia or EGFR activation, PGK1 can translocated in mitochondria to activate PDHK1 phosphorylation, suppressing the TCA cycle ultimately promoting tumour growth.^14^ Same in brain tumour, hypoxia induced mTOR‐mediated PGK1 acetylation to induce autophagy.^36^ Therefore, the regulatory mechanisms of PGK1 under hypoxia conditions may be more complex than currently understood. These complexities render the molecular mechanisms underpinning tumour cell adaptation to hypoxia both multifaceted and inadequately understood. However, the results of our experiments confirmed that, the HIF‐1α‐PGK1 axis remains the critical regulatory mechanism under hypoxic conditions in ESCC cells. Our findings, supported by ChIP assays and luciferase reporter assays, confirm that HIF‐1α transcriptionally activates PGK1 expression under hypoxia, and this pathway is central to PGK1's role in ESCC progression. Gaining a better understanding of these mechanisms is essential for improving our knowledge of tumour biology and has great potential for guiding the creation of tailored treatments meant to stop hypoxia‐driven PGK1 upregulated esophageal cancer processes.

Thus, PGK1 accumulation brought on by hypoxia‐induced HIF‐1α expression in esophageal cancer may be one of the molecular mechanisms underlying EMT and stemness‐supporting transcriptions, which in turn promote tumour progression, metastasis, and recurrence, ultimately leading to a poor prognosis for patients with esophageal cancer. Therefore, our research indicates that PGK1 has biological and clinical value since it may be employed as a target for ESCC treatment as well as a predictor of recurrence and metastasis.

## AUTHOR CONTRIBUTIONS

J.C.X and L.F.W. wrote the manuscript. J.C.X., L.F.W. and T.Y.C. carried out the experimental work. J.C.X., T.Y.C., Y.F.Z. and L.Y.B. conducted the animal experiments. J.C.X., L.F.W., and T.Y.C. revised the manuscript. Y.Q.Z. and P.H.Z. designed the experiments and supervised the projetc. All the authors have approved the final version of the manuscript.

## CONFLICT OF INTEREST STATEMENT

The authors declare that they have no known competing financial interests or personal relationships that could have appeared to influence the work reported in this paper.

## ETHICS STATEMENT

The study was approved by the Ethics Review Committee of Zhongshan Hospital, Fudan University, and written informed consent was obtained from all patients for their data and samples to be used for research purposes. The animal experiments were conducted in accordance with the guidelines set forth by the Institutional Animal Care and Utilization Committee of Zhongshan Hospital, Fudan University. Every effort was made to minimise the number and suffering of the included animals.

## Supporting information



Supporting Information

## Data Availability

All data supporting the findings of this study are available within the article and its supplementary information file or from the corresponding author upon request.
