# Supplementary material for: Hypoxia‐induced PGK1 expression promotes esophageal squamous cell carcinoma progression via stimulating MYH9‐mediated GSK3β/β‐catenin signalling
Source: Clin Transl Med. 2025 Jun 18;15(6):e70376. doi: 10.1002/ctm2.70376 (PMC12177104; doi:10.1002/ctm2.70376)
Supplement: Supplementary file 1 — Supporting Information [file CTM2-15-e70376-s001.docx]

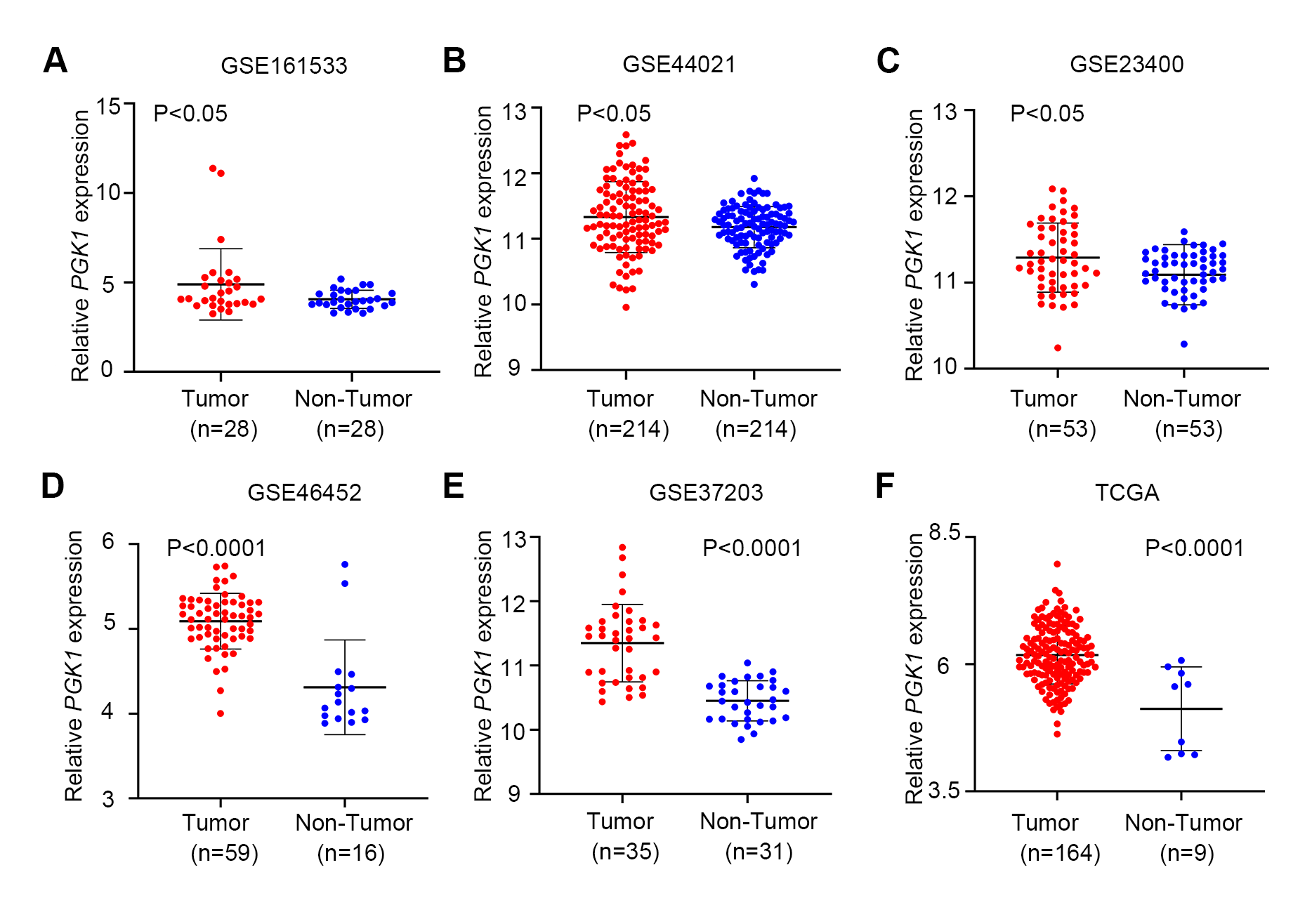


**Figure. S1. Database analysis of PGK1 expression between tumor and not-tumor in database ESCC clinical samples.** GSE161533 (A), GSE44021 (B), GSE23400 (C), GSE46452 (D), GSE37203 (E) and TCGA (F).


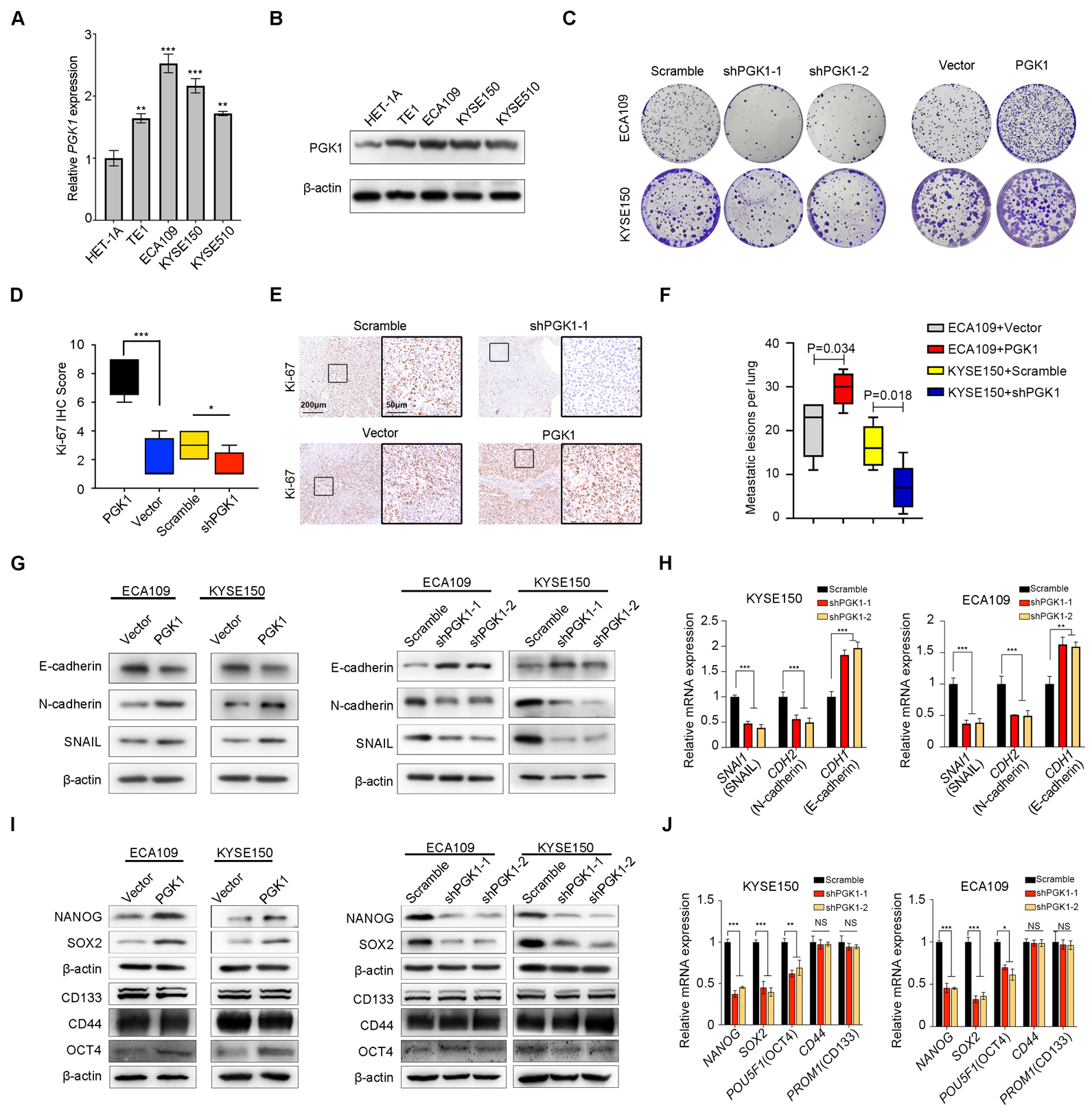


**Figure S2. PGK1 promotes tumorigenicity, and migration ability of ESCC cells.**

(A-B) PGK1 expression levels in esophageal cancer cell lines (ECA109, KYSE150, KYSE510, TE1) and normal esophageal cell lines (HET-1A) measured by qRT-PCR (A) and Western blot (B). Data are presented as mean ± SD; ***P* < 0.01, ****P* < 0.001. (C) Colony formation assay demonstrating the effect of PGK1 on colony formation in ESCC cells. (D) Ki-67 immunohistochemistry (IHC) score in xenograft tumors formed by ECA109 cells with PGK1 overexpression or knockdown. Data are presented as mean ± SD; **P* < 0.05, ****P* < 0.001. (E) Representative Ki-67 expression from mice injected with ESCC cells overexpressing or knocking down PGK1. (F) Metastasis lesions from *in vivo* lung metastasis models. (G) Western blot analysis of EMT markers in ECA109 and KYSE150 cells with PGK1 overexpression and knockdown. (H) qRT-PCR analysis of EMT markers (*CDH1, CDH2, SNAI1*) in KYSE150 and ECA109 cells with PGK1 knockdown. Data are presented as mean ± SD; ***P* < 0.01, ****P* < 0.001, NS = not significant. (I) Western blot analysis of stemness-related markers in ECA109 and KYSE150 cells with PGK1 overexpression (left) or knockdown (right). (J) qRT-PCR analysis of stemness-related markers (*NANOG, SOX2, POU5F1, CD44, PROM1*) in KYSE150 and ECA109 cells with PGK1 knockdown. Data are presented as mean ± SD; **P* < 0.05, ***P* < 0.01, ****P* < 0.001, NS = not significant.

**
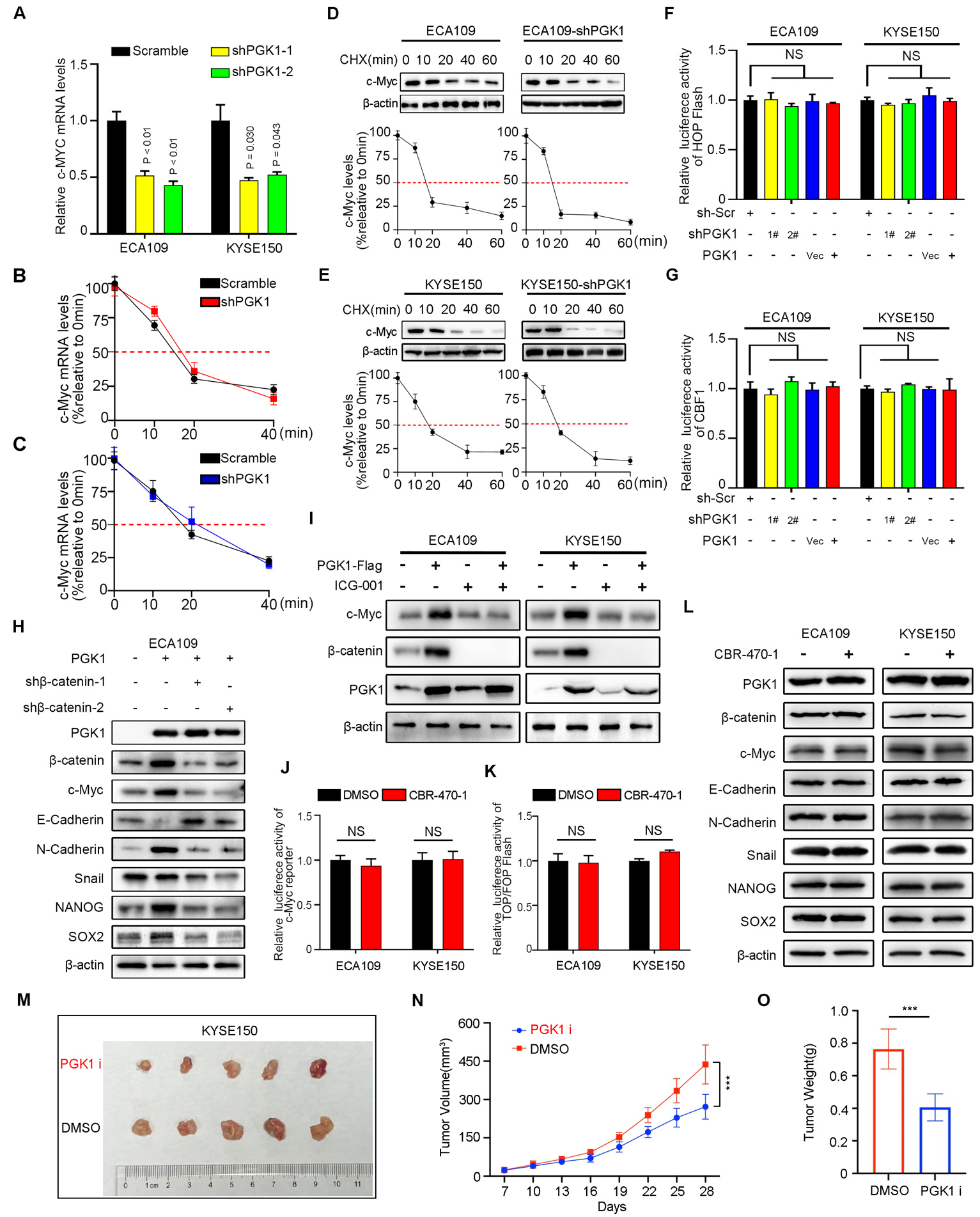
**

**Figure S3. PGK1 enhances the transcription of c-Myc via activating β-catenin pathway.**

(A) qRT-PCR analysis of c-Myc mRNA levels in ECA109 and KYSE150 cells with PGK1 knockdown (shPGK1-1, shPGK1-2). Data are presented as mean ± SD; ***P* < 0.01, ****P* < 0.001. (B-C) Stability of c-Myc mRNA measured in ECA109 (B) and KYSE150 (C) cells treated with actinomycin D at various time points (10, 20, 30, 40 minutes). (D-E) Cycloheximide (CHX) chase assays showing c-Myc protein stability in ECA109 (D) and KYSE150 (E) cells with PGK1 knockdown. Representative Western blots and quantification of c-Myc protein levels (% relative to time 0) are shown. (F-G) Luciferase reporter assays showing transcriptional activity of HOP-Flash (F) and CBF1-responsive element (G) in ECA109 and KYSE150 cells with PGK1 knockdown. Data are presented as mean ± SD; NS = not significant. (H) Western blot analysis of β-catenin and EMT/stemness markers (E-cadherin, N-cadherin, Snail, NANOG, SOX2) in ECA109 cells with PGK1 and β-catenin knockdown (shβ-catenin-1, shβ-catenin-2). (I) Western blot analysis of β-catenin and c-Myc protein levels in ECA109 and KYSE150 cells with PGK1 overexpression treated with β-catenin inhibitor ICG-001. (J-K) Dual-luciferase reporter assays showing c-Myc transcriptional activity (J) and β-catenin transcriptional activity (K) in ECA109 and KYSE150 cells treated with PGK1 metabolic enzyme inhibitor CBR-470-1. Data are presented as mean ± SD; NS = not significant. (L) Western blot analysis of β-catenin, c-Myc, and EMT/stemness markers (E-cadherin, N-cadherin, Snail, NANOG, SOX2) in ECA109 and KYSE150 cells treated with CBR-470-1. (M-O) In vivo tumorigenicity assay using KYSE150 cells with or without PGK1-i(NG52). (M) Representative images of tumors. (N) Tumor volume measured over time. (O) Quantification of tumor weights (P <0.001). Data are presented as mean ± SD.


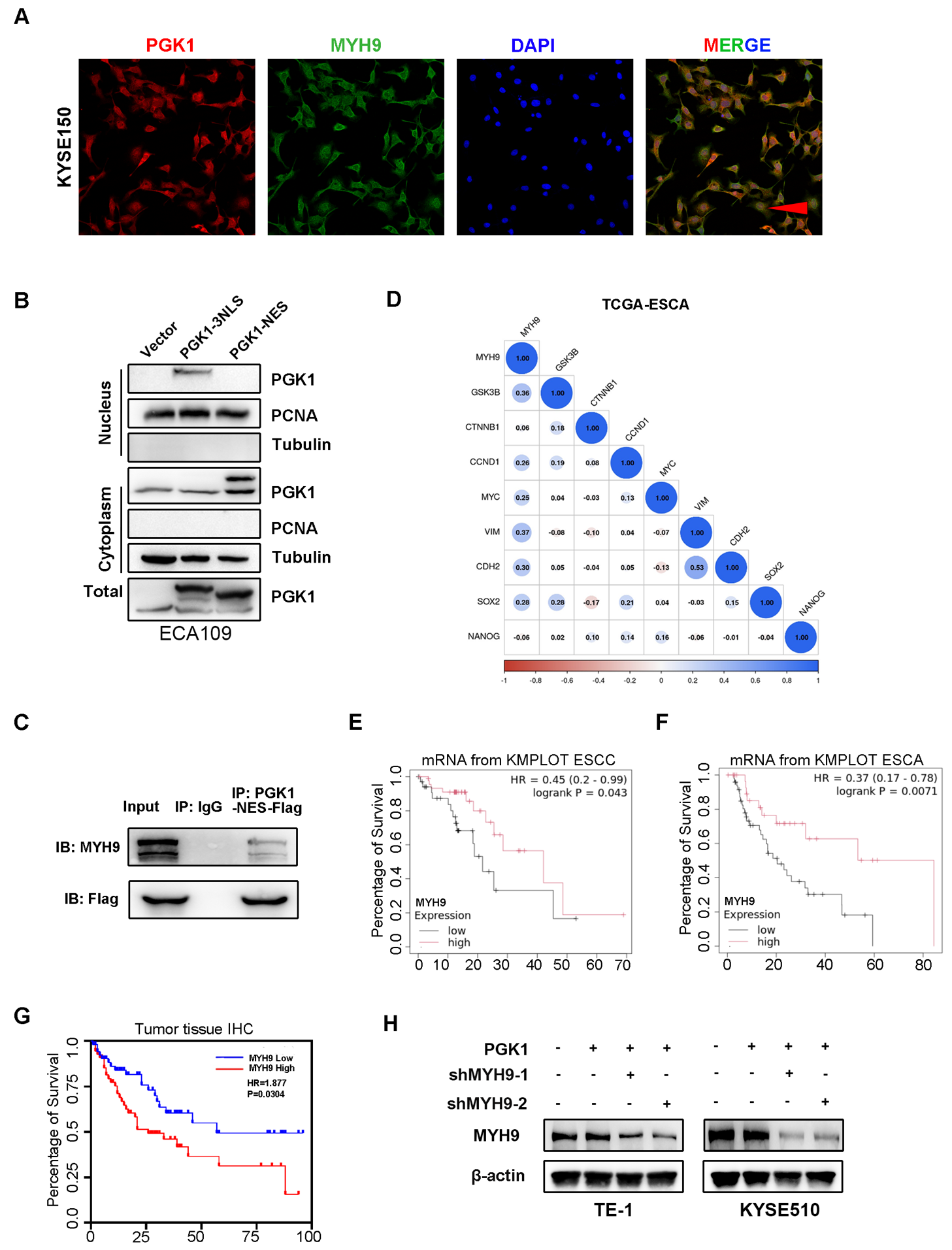


**Figure S4. Subcellular localization of PGK1 and clinical correlation between MYH9-PGK1-targeted genes and MYH9-ESCC-patients.**

(A) Representative images of immunofluorescence staining. (B) Western blot analysis showing nuclear and cytoplasmic localization of PGK1 in ECA109 cells. PGK1-3NLS and PGK1-NES constructs were used to confirm nuclear and cytoplasmic localization, respectively. (C) Co-IP analysis of and PGK1-NES construct confirms its interaction with MYH9 in ECA109 cell. (D) Correlation matrix of *MYH9*, and related signaling molecules (*GSK3β, CTNNB1, CCND1, MYC, VIM, CDH2, SOX2, NANOG*) in the TCGA-ESCA dataset. Positive correlations are represented in blue, and negative correlations in red. Correlation coefficients (R) are shown. (E-F) Kaplan-Meier survival curves of ESCC patients (E), ESCA (F) patients from the KMplot database and stratified by MYH9 expression levels. Hazard ratios (HR) and log-rank P-values are indicated. (G) Survival analysis based on IHC results from tumor tissues (n=108). (H) Western blot analysis of c-Myc in TE-1 and KYSE510 cells after MYH9 knockdown with or without PGK1 overexpression.


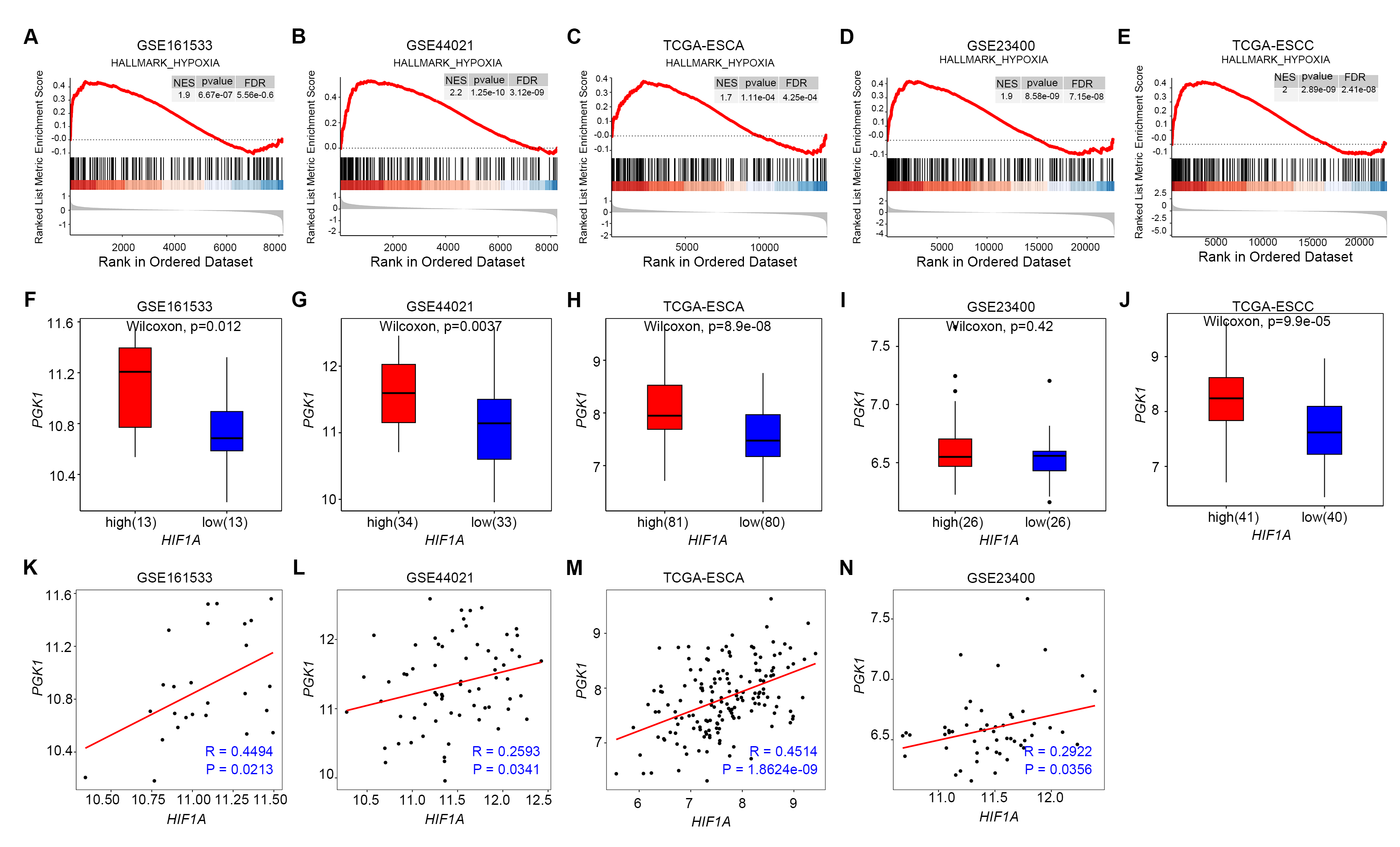


**Figure. S5. Database analysis of PGK1 and HIF-1α** **expression relationship between tumor and not-tumor in database ESCC clinical samples.** (A-E) Gene Set Enrichment Analysis (GSEA) plots showing enrichment of the hypoxia pathway in datasets GSE1615333 (A), GSE44021 (B), TCGA-ESCA (C), GSE23400 (D), and TCGA-ESCC (E) with high PGK1 expression. The normalized enrichment scores (NES) and false discovery rate (FDR) values are indicated. (F-J) Boxplots showing PGK1 expression in esophageal cancer samples with high versus low HIF-1α expression in GSE1615333 (F), GSE44021 (G), TCGA-ESCA (H), GSE23400 (I), and TCGA-ESCC (J). P-values were calculated using the Wilcoxon rank-sum test. (K-O) Scatter plots illustrating the correlation between HIF-1α and PGK1 expression levels in datasets GSE1615333 (K), GSE44021 (L), TCGA-ESCA (M), and GSE23400 (N). Pearson correlation coefficients (R) and P-values are indicated.


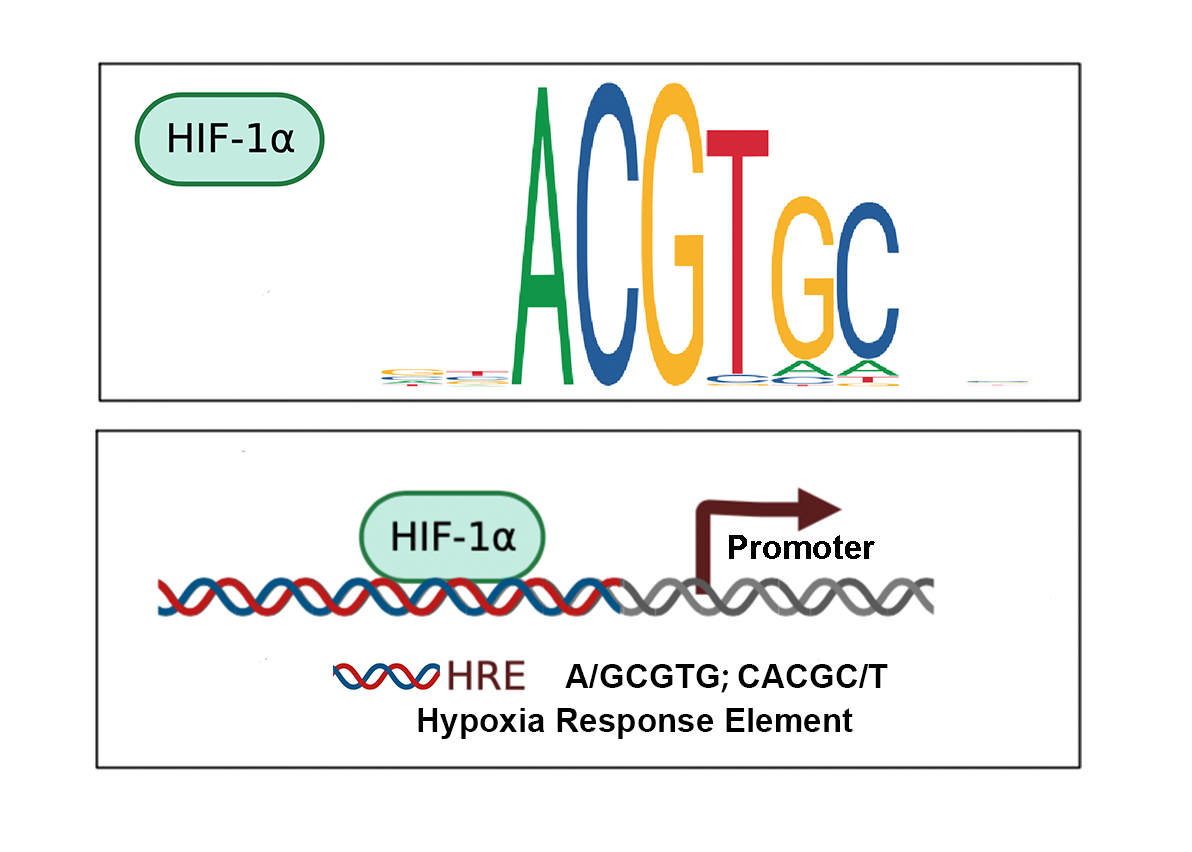


**Figure S6. HIF-1α binding motif and hypoxia response element (HRE) in the PGK1 promoter.**

(Top) The consensus binding motif of HIF-1α derived from the analysis of hypoxia-responsive genes. The motif logo depicts the conserved sequence, highlighting the key bases in the ACGTG core sequence.

(Bottom) Schematic representation of HIF-1α binding to the hypoxia response element (HRE) within the PGK1 promoter region. The canonical HRE sequence (A/GCGTG; CACGC/T) is shown, indicating its role in hypoxia-induced transcriptional activation.

**Table. S1. Association between PGK1 expression and clinicopathological features of patients with esophageal cancer(ESCC)**

|  |  | PGK1 expression | |  |  |
| --- | --- | --- | --- | --- | --- |
|  | Total | Low | High |  |  |
|  | (n = 108) | (n = 51) | (n = 57) | χ^2^ | *P* value |
| Gender |  |  |  | 4.532 | 0.05697 |
| Male | 82 | 34 | 48 |  |  |
| Female | 26 | 17 | 9 |  |  |
| Age(year) |  |  |  | 1.126 | 0.3864 |
| <65 | 46 | 19 | 27 |  |  |
| ≥65 | 62 | 32 | 30 |  |  |
| Tumor size(cm) | |  |  | 25.016 | **<0.001*** |
| <5 | 53 | 38 | 15 |  |  |
| ≥5 | 55 | 13 | 42 |  |  |
| T staging |  |  |  | 21.704 | **<0.001*** |
| T1-T2 | 25 | 22 | 3 |  |  |
| T3-T4 | 83 | 29 | 54 |  |  |
| Nodal staging |  |  |  | 50.529 | **<0.001*** |
| N0 | 50 | 42 | 8 |  |  |
| N1-3 | 58 | 9 | 49 |  |  |
| Differentiation |  |  |  | 23.614 | **<0.001*** |
| Well | 26 | 22 | 4 |  |  |
| Moderate | 62 | 26 | 36 |  |  |
| Poor | 20 | 3 | 17 |  |  |
| TNM |  |  |  | 53.323 | **<0.001*** |
| I/II | 49 | 42 | 7 |  |  |
| III/IV | 59 | 9 | 50 |  |  |
|  |  |  |  |  |  |

**Table. S2. Association between PGK1 expression and clinicopathological features of patients with esophageal cancer(ESCC)**

|  |  | PGK1 expression | |  |  |
| --- | --- | --- | --- | --- | --- |
|  | Total | Low | High |  |  |
|  | (n = 108) | (n = 51) | (n = 57) | χ^2^ | *P* value |
| TNM |  |  |  | 55.551 | **<0.001*** |
| I Stage  II Stage | 14  35 | 14  28 | 0  7 |  |  |
| III Stage  IV Stage | 53  6 | 9  0 | 44  6 |  |  |
| N Stage |  |  |  | 24.72 | **<0.001*** |
| N0-N1 | 86 | 51 | 35 |  |  |
| N2-N3 | 22 | 0 | 22 |  |  |
| Advanced Type | |  |  | 2.384 | **0.497** |
| Medullary  Constricting | 61  5 | 28  2 | 33  3 |  |  |
| Fungating  Ulcerative | 32  10 | 14  7 | 18  3 |  |  |

| **Table. S3. Cox's proportional hazards model analysis of prognostic factors in patients with esophageal cancer (ESCC)** | | | |
| --- | --- | --- | --- |
|  |  | Overall survival | |
| Variables | unfavorable/favorable | HR(95%CI) | *P* value |
| **Univariate analysis** |  |  |  |
| PGK1 | high/low or absent | 3.911 (2.465-6.206) | **<0.001*** |
| Gender | female/male | 0.398(0.224-0.709) | **0.002*** |
| Age | ≥65/<65 | 1.115(0.726-0.713) | 0.618 |
| Differentiation | poor/well or moderate | 1.865(1.090-3.192) | **0.023*** |
| Tumor size | ≥5cm /<5cm | 1.775(1.154-2.730) | **0.009*** |
| pN factor | +/- | 1.932(1.247-2.991) | **0.003*** |
| TNM stage | III-IV/ I-II | 1.969 (1.268-3.058) | **0.003*** |
| **Multivariate analysis** |  |  |  |
| PGK1  Gender | high/low or absent  female/male | 8.831(3.740-20.855)  0.351(0.192-0.641) | **<0.001***  **0.001*** |
| Differentiation | poor/well or moderate | 0.961(0.461-2.003) | 0.915 |
| Tumor size | ≥5cm /<5cm | 1.141(0.683-1.906) | 0.613 |
| pN factor | +/- | 1.423(0.194-10.439) | 0.729 |
| TNM stage | III-IV/ I-II | 0.226(0.027-1.872) | 0.168 |
|  | | |  |

**Table. S4. Antibodies Used in the Study and Their Sources**

| Antibody Name | Manufacturer | Catalog Number |
| --- | --- | --- |
| Beta Actin | ProteinTech | 66009-1-Ig |
| PGK1 | ProteinTech | 17811-1-AP |
| E-cadherin | Abcam | Ab40772 |
| N-cadherin  SNAIL  NANOG  SOX2  c-Myc  β-catenin  MYH9  Tubulin  PCNA  Mouse IgG  Rabbit IgG  Flag  GSK3β  p-GSK3β(Ser9)  Ubiquitin  HIF-1α(IHC)  HIF-1α  c-Myc(IHC) | Abcam  ProteinTech  ProteinTech  ProteinTech  ProteinTech  Abcam  ProteinTech  ProteinTech  ProteinTech  ProteinTech  ProteinTech  Sigma  CST  ProteinTech  ABclonal  ProteinTech  CST  Santa | Ab18203  6183-1-AP  14295-1-AP  11064-1-AP  10828-1-AP Ab32572  11128-1-AP  11224-1-AP  10205-2-AP  SA00001-1  SA00001-2  F1804  12456  67558-1-Ig  A19686  20960-1-AP  36169S  Sc-40 |
| CD44  CD133  OCT4  Lamin A/C | CST  ProteinTech  ProteinTech  ProteinTech | 37259  18470-1-AP  11263-1-AP  10298-1-AP |

**Table S5. Primers and oligonucleotides sequences.**

| **Interfering oligonucleotides** | |
| --- | --- |
| sh-Human PGK1-1 | GCUUCUGGGAACAAGGUUATTUAACCUUGUUCCCAGAAGCTT |
| sh-Human PGK1-2 | CCAAGUCGGUAGUCCUUAUTTAUAAGGACUACCGACUUGGTT |
| sh-Human β-catenin-1 | AACAGTCTTACCTGGACTCTG |
| sh-Human β-catenin-2 | AAAGGCAATCCTGAGGAAGAG |
| sh-Human MYH9-1 | CAGGCCUGUUCUGUGUGGUCAUCAA |
| sh-Human MYH9-2 | AAGCAGGCGUGCGUGCUCAUGAUAA |
| sh-Human HIF1-A | AAAAGGGAUUAACUCAGUUUGTTGGATCCAACAAACUGAGUUAAUCCC |
| Scramble | TTCTCCGAACGTGTCACGT |
| **PCR primers** |  |
| Human *PGK1* |  |
| Forward primer | GAACAAGGTTAAAGCCGAGCC |
| Reverse primer | GTGGCAGATTGACTCCTACCA |
| Human *SNAI1* |  |
| Forward primer | TCGGAAGCCTAACTACAGCGA |
| Reverse primer | AGATGAGCATTGGCAGCGAG |
| Human *TWIST1* |  |
| Forward primer | CACCATTGGCAATGAGCGGTTC |
| Reverse primer | AGGTCTTTGCGGATGTCCACGT |
| Human *NANOG* |  |
| Forward primer | GTCCGCAGTCTTACGAGGAG |
| Reverse primer | GCTTGAGGGTCTGAATCTTGCT |
| Human *SOX2* |  |
| Forward primer | GCCGAGTGGAAACTTTTGTCG |
| Reverse primer | GGCAGCGTGTACTTATCCTTCT |
| Human *CD44* |  |
| Forward primer | CCAGAAGGAACAGTGGTTTGGC |
| Reverse primer | ACTGTCCTCTGGGCTTGGTGTT |
| Human *POU5F1*  *（OCT4）* |  |
| Forward primer | CCTGAAGCAGAAGAGGATCACC |
| Reverse primer | AAAGCGGCAGATGGTCGTTTGG |
| Human *PROM1（CD133）* |  |
| Forward primer | AGTCGGAAACTGGCAGATAGC |
| Reverse primer | GGTAGTGTTGTACTGGGCCAAT |
| Human *MYC* |  |
| Forward primer | CCTGGTGCTCCATGAGGAGAC |
| Reverse primer | CAGACTCTGACCTTTTGCCAGG |
| Human *ACTB* |  |
| Forward primer | CACCATTGGCAATGAGCGGTTC |
| Reverse primer | AGGTCTTTGCGGATGTCCACGT |
| **CHIP primers** |  |
| PGK1 binding motif1 primer | |
| Forward primer | TCTTCGCCGCTACCCTTGTG |
| Reverse primer | CAGGAACAGGGCCCACACTAC |
| PGK1 binding motif2 primer | |
| Forward primer | GGTGTTCCGCATTCTGCAAG |
| Reverse primer | CAGAGCACAGAGAGCACGC |
